# Mitochondrial redox homeostasis links organellar stress surveillance to germline and somatic integrity in *Caenorhabditis elegans*

**DOI:** 10.1016/j.redox.2026.104115

**Published:** 2026-03-09

**Authors:** Marina Valenzuela-Villatoro, Patricia de la Cruz-Ruiz, David Guerrero-Gómez, Eva Gómez-Orte, Alfonso Schiavi, Silvia Maglioni, Mayte Montero, Rosalba I. Fonteriz, José Carlos Casas-Martínez, Nicole E. Briand, Jingxiu Xu, María Jesús Rodriguez-Palero, Marta Artal-Sanz, Katarzyna Olek, Justyna Polaczyk, Michal Turek, Wojciech Pokrzywa, Suhong Xu, Javier E. Irazoqui, Brian McDonagh, Javier Álvarez, María Olmedo, Natascia Ventura, Juan Cabello, Antonio Miranda-Vizuete

**Affiliations:** a Redox Homeostasis Group, Instituto de Biomedicina de Sevilla, IBiS/Hospital Universitario Virgen del Rocío/CSIC/Universidad de Sevilla, Seville, Spain; b Centro de Investigación Biomédica de la Rioja (CIBIR), Logroño, La Rioja, Spain; c IUF-Leibniz Research Institute for Environmental Medicine, Duesseldorf, Germany; d Institute for Clinical Chemistry and Laboratory Diagnostic, Medical Faculty, Heinrich Heine University, Duesseldorf, Germany; e Departamento de Bioquímica y Biología Molecular y Fisiología, Facultad de Medicina, Universidad de Valladolid, Valladolid, Spain; f Unidad de Excelencia Instituto de Biomedicina y Genética Molecular (IBGM), Universidad de Valladolid y Consejo Superior de Investigaciones Científicas (CSIC), Valladolid, Spain; g Discipline of Physiology, School of Pharmacy and Medical Sciences, University of Galway, Galway, Ireland; h Apoptosis Research Centre, University of Galway, Galway, Ireland; i Galway RNA Research Cluster, University of Galway, Galway, Ireland; j Department of Microbiology, UMass Chan Medical School, Worcester, MA, USA; k International Biomedicine-X Research Center of the Second Affiliated Hospital, Zhejiang University School of Medicine and the Zhejiang University-University of Edinburgh Institute, Haining, Zhejiang, China; l Center for Stem Cell and Regenerative Medicine and Department of Burn and Wound Repair of the Second Affiliated Hospital, Zhejiang University School of Medicine, Hangzhou, Zhejiang, China; m School of Basic Medical Sciences, Zhejiang University School of Medicine, Hangzhou, Zhejiang, China; n Andalusian Centre for Developmental Biology, Consejo Superior de Investigaciones Científicas (CSIC), Universidad Pablo de Olavide, Junta de Andalucía, Department of Molecular Biology and Biochemical Engineering, Avenida Rectora Rosario Valpuesta 1, Dos Hermanas, Seville, Spain; o Departamento de Biociencias, Facultad de Ciencias de la Salud, Universidad Loyola Andalucía, Avda. de las Universidades s/n, Dos Hermanas, Sevilla, Spain; p Laboratory of Protein Metabolism, International Institute of Molecular and Cell Biology in Warsaw, Warsaw, Poland; q Laboratory of Animal Molecular Physiology, Institute of Biochemistry and Biophysics, Polish Academy of Sciences, Warsaw, Poland; r Departamento de Genética, Facultad de Biología, Universidad de Sevilla, Avenida Reina Mercedes s/n, Seville, Spain; s Institute of Cell Biology, Heinrich Heine University Duesseldorf, Universitaetsstrasse 1, Duesseldorf, Germany; t Department for the Promotion of Human Science and Quality of Life, University of Rome San Raffaele, Via di Val Cannuta 247, Rome, Italy

**Keywords:** Mitochondria, Elegans, Thioredoxin reductase, Glutathione reductase, Unfolded protein response, ATFS-1

## Abstract

Mitochondrial redox homeostasis is essential for cellular metabolism and organismal development. To investigate the consequences of disrupting redox homeostasis in this organelle in a metazoan organism, we generated a double mutant lacking mitochondrial glutathione reductase (*gsr-1a*) and thioredoxin reductase (*trxr-2*) genes in *Caenorhabditis elegans*. While *gsr-1a* or *trxr-2* single mutants are phenotypically normal, double *gsr-1a trxr-2* mutants displayed small body size, gonadal migration defects, reduced brood size, and prolonged egg-laying period, without developmental delay or lethality. Transcriptomic analysis revealed strong induction of ATFS-1-dependent stress and detoxification genes. Consistent with this, *gsr-1a trxr-2* worms exhibited constitutive ATFS-1 nuclear localization and robust *Phsp-6::gfp* expression. Triple *gsr-1a trxr-2; atfs-1* mutants were nonviable, demonstrating that unfolded protein response (UPR^mt^) activation is essential under mitochondrial redox stress.

Despite the induction of a stress response at the transcriptional level, *gsr-1a trxr-2* double mutants were not more resistant to oxidative or pathogen stressors. Moreover, these mutants maintained normal respiration, ATP and ROS production while displaying altered mitochondrial morphology in a tissue-specific manner, independent of mitophagy genes but dependent on mitochondrial fission or fusion machinery. Functionally, *gsr-1a trxr-2* mutants showed impaired motility, reduced calcium uptake upon carbachol stimulation, enhanced hypodermal wound repair, and decreased fertilization efficiency associated with lower muscle exopher production.

Overall, our data show that simultaneous loss of mitochondrial GSR-1a and TRXR-2 compromises growth, fertility and muscle performance and triggers a constitutive ATFS-1-dependent UPR^mt^ that sustains viability revealing mitochondrial redox control as a core determinant of organismal proteostasis.

## Highlights

• *gsr-1a trxr-2* double mutants are viable but show small size, gonad migration defects and reduced progeny.
• Loss of both reductases in mitochondria triggers a constitutive ATFS-1–dependent UPR^mt^.
• ATFS-1 is essential for *gsr-1a trxr-2* worms survival.
• *gsr-1a trxr-2* animals remodel mitochondrial morphology in a tissue-specific manner.
• *gsr-1a trxr-2* double mutants exhibit impaired muscle and sperm function but enhanced wound healing.

## Graphical abstract

**Figure:**
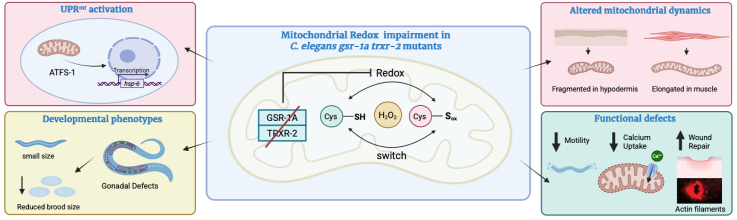

## 1 Introduction

Mitochondria are subcellular organelles that not only play key roles in cellular function such as ATP synthesis, maintenance of Ca^2+^ homeostasis, Fe–S cluster biogenesis, heat production or lipid synthesis but are also a central hub for intracellular communication either by releasing signaling molecules or by direct interaction with other organelles to cope with cellular stress and metabolic demands [1]. To coordinate all these functions, mitochondria have developed a remarkable plasticity to form dynamic organellar tubular networks that are exquisitely controlled by fusion and fission mechanisms [2].

When mitochondrial function is compromised, for example by respiration inhibition, mitochondrial DNA damage, impairment of protein import or proteotoxic stress within the mitochondrial matrix, a protective response is initiated to counteract the insult, that mainly operates through two primary defense mechanisms. The first one acts at the molecular level and consists of a retrograde signal from mitochondria to the nucleus leading to the coordinated expression of a network of mitochondrial proteases, chaperones and membrane transporters, collectively known as the mitochondrial unfolded protein response (UPR^mt^), which is orchestrated by the transcription factor ATF5 in mammals or ATFS-1 in the nematode *C*. *elegans* [[3], [4], [5]]. When UPR^mt^ fails to cope with the mitochondrial insult, a second defense mechanism at the complete organelle level is triggered, by which the mitochondrial network is reorganized by fusion and fission mechanisms and mitophagy is activated to remove irreversibly damaged mitochondria with the ultimate objective of maintaining a healthy mitochondrial population [6,7]. Notably, in some cell types such as neurons and muscle cells, damaged mitochondria may also be extruded from the cell in large extracellular vesicles known as exophers, providing an additional route for clearing dysfunctional organelles beyond canonical mitophagy [[8], [9], [10]].

Because mitochondria are the main site of reactive oxygen species (ROS) production, the mitochondrial redox status needs to be tightly controlled to prevent macromolecular damage such as oxidative misfolding by excessive ROS production (oxidative distress). At the same time, mitochondria must maintain a balanced redox-dependent signaling network to ensure proper intracellular communication and specific posttranslational modifications (oxidative eustress) [11,12]. For this purpose, mitochondria are equipped with dedicated ROS detoxifying enzymes such as superoxide dismutases, glutathione peroxidases or peroxiredoxins but at the same time they also have enzymatic networks to regulate the redox balance like the thioredoxin and glutathione/glutaredoxin systems which use NADPH as source of reducing power [[12], [13], [14]].

While initially considered as separate pathways, the cooperation between the thioredoxin and glutathione/glutaredoxin systems is now well established across species. For instance, in yeast lacking glutathione reductase, oxidized glutathione (GSSG) reduction is carried out by the cytoplasmic thioredoxin system composed of one thioredoxin reductase and two thioredoxins [15,16]. In *C. elegans*, cytoplasmic thioredoxin reductase TRXR-1 is functionally redundant with glutathione reductase GSR-1 to allow a proper molting progression [17]. However, TRXR-1 is unable to rescue the embryonic lethality of *gsr-1* null mutants [18], demonstrating developmental or tissue-specific requirements for this redundancy in the cytoplasm. In mice, mutations in the genes encoding the components of the cytoplasmic and mitochondrial thioredoxin systems result in embryonic lethal phenotypes [[19], [20], [21], [22]] while mutant mice lacking glutathione reductase are viable with very mild phenotypes [23]. Evidence for a functional redundancy between the two systems in the cytoplasm arises from studies in mice hepatocytes lacking both thioredoxin reductase and glutathione reductase that remain viable by boosting glutathione (GSH) *de novo* synthesis through the transsulfuration pathway [24,25]. This functional redundancy is not restricted to animals but also includes plants, as *Arabidopsis thaliana* defective in both cytoplasmic thioredoxin reductase NTRA and glutathione reductase GR1 enzymes are not viable with a pollen lethal phenotype because of their inability to fertilize wild-type ovules [26].

In contrast with the functional redundancy of the cytoplasmic thioredoxin and glutathione systems, less is known on their cooperation within mitochondria. Indeed, to our knowledge, this redundancy has only been shown in yeast and *Arabidopsis thaliana*. In *Saccharomyces cerevisiae* and *Schizosaccharomyces pombe*, the absence of mitochondrial glutathione reductase causes a major disruption of cellular redox homeostasis which is only partly alleviated by the mitochondrial thioredoxin system [27,28] while in *Arabidopsis thaliana* the mitochondrial thioredoxin system and the GSSG exporter ATM3 coordinate to back up the absence of mitochondrial glutathione reductase GR2 [29]. However, it is not known whether this functional redundancy operates in metazoan.

To fill this gap, we have approached the question using the nematode *C*. *elegans*, a well-established model organism, taking advantage of its genetic tractability, high conservation of redox signaling pathways and its transparency throughout all its developmental stages, allowing *in vivo* imaging approaches [30]. In this work, we investigate whether the mitochondrial thioredoxin and glutathione systems exhibit functional redundancy in a metazoan and show that concurrent loss of mitochondrial *trxr-2* and *gsr-1a* disrupts redox homeostasis and elicits a strong ATFS-1–dependent UPR^mt^ that is essential for organismal viability. Double mutants exhibit reduced body size, defects in gonadal migration, and compromised fertility, revealing key physiological processes sensitive to mitochondrial redox imbalance. Moreover, we uncover tissue-specific alterations in mitochondrial dynamics that impair muscle performance, hypodermal repair responses, and sperm function.

Together, our findings define the phenotypic landscape resulting from combined disruption of mitochondrial thioredoxin and glutathione systems in an animal model organism, providing a framework for future mechanistic dissection of mitochondrial redox control as a critical determinant of cellular and organismal proteostasis.

## 2 Materials and methods

### 2.1 *C. elegans* strains

The standard methods used for culturing and maintenance of *C. elegans* were as previously described [31]. A list of all strains used and generated in this study is provided in Supplementary Table 1. The *gsr-1a(syb266)* allele was generated at SunyBiotech (https://www.sunybiotech.com/) by CRISPR-Cas9 editing using the following sgRNAs: AMV04-sg1, 5′-cccttctgaATGCTCCGATTTCG-3′; AMV04-sg2, 5′-ccttctgaATGCTCCGATTTCGC-3′; AMV04-sg3, 5′-CCGATTTCGCTGCATTTTGAGCA-3′; AMV04-sg4, 5′-GCTGCTTTTGAGCACTTCGAGg-3′. The oligo repair template was: 5′-gtttattttttgctgatcaaatgtctaaaaacttctgtgaacccttctga-gtattaaattgttactttttggtttaaaaaaagtttttgtagtctttgtg-3′.

All VZ strains are 6x outcrossed with N2 wild type, except those strains generated by CRISPR-Cas9 (*syb* alleles) that were 2x outcrossed. Worm reagents and details on the protocols used for genotyping the different alleles reported in this work can be provided upon request. Unless otherwise noted, all experiments were performed on synchronized worms generated by allowing 10 to 15 gravid hermaphrodites to lay eggs during two to 3 h on seeded plates at 20 °C.

### 2.2 Size, gonad migration defects and oocyte quantification

Quantification was carried out in animals grown for 72 and 96 h at 20 °C after synchronized egg-lay. Worms were paralyzed with 10 mM levamisole (Sigma-Aldrich, Cat. #L9756) on a microscope slide in an Olympus BX61 fluorescence microscope equipped with a DP72 digital camera coupled to CellSens Software. Images were acquired and the length and area of the animals were determined using ImageJ software [32]. Quantification of gonad migration defects and the number of oocytes was directly performed on immobilized animals at the microscope. The number of oocytes corresponds to the proximal arm of the posterior gonad.

### 2.3 Developmental timing

We measured developmental timing of single worms using a bioluminescence-based method previously described [33] but using the reporter *sevIs1 [Psur-5::luc+::GFP]* [34]. To obtain age-synchronized eggs, we transferred 10-15 hermaphrodites to a fresh NGM plate and allowed them to lay eggs for 1 h. Synchronized eggs were individually placed in wells of a 96 well-plate containing 100 μl of S-basal plus 10 μg/ml cholesterol (Sigma-Aldrich, Cat. #C8667) with 200 μM D-Luciferin (Biothema BT11100). After placing all embryos, 100 μl of S-basal with 20 g/l E*. coli* OP50-1 was added to each well. Samples from different strains were alternated on the plate to avoid local effects. Plates were sealed with a gas-permeable membrane and transferred to a luminometry reader (Berthold Centro XS3) placed inside a cooled incubator (Panasonic MIR-254). Luminescence was recorded for 1 s per well, at 5-min intervals.

### 2.4 Embryonic arrest and brood size quantification

For embryonic arrest, 10 gravid hermaphrodites were allowed to lay eggs for 1 h in three replica plates. After removal of the parents, the embryos were counted and allowed to develop three days at 20 °C. Viable progeny was quantified and detracted to the number of laid embryos to determine the embryonic arrest. Brood size was quantified from 20 L4 hermaphrodites, placed in groups of five animals on four separate plates. Worms were transferred to fresh plates every 24 h, and the number of viable progeny was recorded daily for a total of eight days. The total brood size was calculated as the sum of all viable progeny by each worm throughout the experimental period.

### 2.5 RNAseq and q-PCR

Worms were synchronized using bleaching solution (50 % dH_2_O (v/v), 30 % commercial NaClO (v/v) and 20 % NaOH (5 N) (v/v)) and grow until young adults. For total RNA extraction, young adult worms were lysed using mirVana Kit (Ambion, Cat. #1560), following manufacturer's instructions. Homogenization of the lysate was performed using a conventional rotor-stator homogenizer polytron, pre-chilled with liquid nitrogen. Global gene expression profile was analyzed at the Genomic Platform of the CIBIR (http://cibir.es/es/plataformas-tecnologicas-y-servicios/genomica-y-bioinformatica). Expression analysis was performed as described [35] using DESeq2 [36] and edgeR [37] algorithms.

For q-PCR analysis, RNA was treated with DNAse to eliminate any DNA contamination. In each sample, a total reaction of 10 μl contained: 500 ng RNA, 1 μl RNase-Free DNAse (Promega, Cat. #M6101), 1 μl 10x Reaction Buffer and DEPC water. Reactions were incubated for 30 min at 37 °C and then stopped by adding 1 μl STOP solution (Promega, Cat. #M6101) and further incubation for 15 min at 65 °C. cDNA synthesis was performed using SuperScript III First-Strand Synthesis System for RT-PCR (Invitrogen, Cat. #18080051) following instructions for random hexamers primers. cDNA was eluted in TE buffer. q-PCR analysis Power SYBR Green Master Mix (ThermoFisher Scientific, Cat. #4367659) and specific primers were used in a QuantStudio 5 Real Time PCR System (Applied Biosystems, ThermoFisher). Normalization to actin *act-1* expression was used to calculate relative expression. The experiments were carried out in three independent replicas. Primers pairs (5′->3′) used were: *msp-76* Fw1: TGACAAGCACACCTACCACA, *msp-76* Rv1: TCCTTTGGGTCGAGAACTCC; *clec-4* Fw1: TCCACAATACCGCCTCAACT, *clec-4* Rv-1: CACGTCCGCCAAATGTCATA; *cyp-14A4* Fw1: TCACAAGCCACCAGAGTCAT, *cyp-14A4* Rv1: CGCGGAGGATTTTCAGTGAG; *ugt-19* Fw1: TCCAGTCGCCTTGATCATGT, *ugt-19* Rv1: TTGCCACATAGTCATCCGGT; *F22B3.7* Fw1: AAATGATGCTGGTATGGGGC, *F22B3.7* Rv1: CCTTCCAAGCCATCACACAC; *act-1* Fw1:CCAGGAATTGCTGATCGTATG, *act-1* Rv1: GGAGAGGGAAGCGAGGATAG.

### 2.6 Fluorescence and light microscopy imaging

Brightfield, differential interference contrast (DIC), and fluorescence images were acquired using an Olympus BX61 fluorescence microscope equipped with a DP72 digital camera and CellSens software for image acquisition and analysis. First day adult worms were mounted on 2% agar pads and anesthetized with 10 mM levamisole prior imaging. For mitophagy quantification using the *Is [Pmyo-3::tomm-20::Rosella]* transgene [38], FITC and TRITC fluorescence filters were used sequentially to calculate the GFP/DsRed fluorescence intensity ratio at 20x magnification. Images were analyzed using ImageJ Software [32].

### 2.7 Confocal imaging

High-resolution confocal imaging was performed using a Nikon A1R confocal microscope equipped with a Plan Apo VC 60x/1.4 NA oil immersion objective. Image acquisition was conducted using the NIS-Elements software and the ND Acquisition module. All Animals were mounted on 2% agar pads and anesthetized with 10 mM levamisole prior to imaging except for mitochondrial structure.

For the analysis of mitochondrial structure in muscle cells using the *wacIs14[Pmyo-3::tomm-20_1-50aa::attB5::mgfp]* transgene [10]) and hypodermal tissue with the *juEx4796 [Pcol-19::mito::gfp; Pttx-3::rfp]* transgene [39]), first day adult worms were immobilized on 5% agarose pads using polystyrene nanoparticles (Alpha Aesar, polystyrene latex microspheres, 0.10 μm, 2.5% w/v dispersion in water, Cat. #42712). Muscle tissue was imaged using Z-stack acquisition followed by maximal intensity projection. For hypodermal tissue, a single focal plane was selected for analysis. Imaging was performed using the same microscope setup as described above. Automated image processing was carried out using a custom ImageJ macro developed by Alejandro Campoy (Microscopy Facility, CABD). The pipeline included background subtraction, median filtering, and automatic thresholding to generate binary images. Mitochondrial features, such as elongation, circularity, and the percentage of area occupied, were quantified according to previously described methods [40].

To quantify ATFS-1 subcellular localization using the *mskEx1 [Patfs-1::atfs-1::gfp]* transgene [4], full-body images of L4 larvae were acquired using a single focal plane and tiled XY scans were used to generate representative images. Imaging parameters were optimized to enable whole-organism reconstruction, capturing both fluorescence and differential interference contrast (DIC) channels. Worms that displayed obvious nuclear labeling in at least 2 intestinal cells were considered as positive for ATFS-1 nuclear localization.

For the quantification of sperm cells in the spermatheca with the *oxIs318 [Pspe-11::mCherry::histone]* transgene [41], sperm nuclei were imaged using Z-stack acquisition followed by maximal intensity projection and saved as 8 bits images. The sperm cells nuclei were quantified using ImageJ wand tool for each spermatheca.

For hyp7 nuclei quantification using the *madEx16 [Pdpy-7::4xNLS::GFP]* transgene [42], a 20x oil-immersion objective was used to image entire animals. Z-stack images were acquired and processed using maximum intensity projection to quantify nuclei from the posterior part of the animals on a single lateral side.

### 2.8 MitoTracker Deep Red assay

Mitochondrial staining was performed using MitoTracker™ Deep Red FM (Invitrogen, Cat. #M22426). A 1 mM stock solution was prepared in DMSO (Panreac, Cat. #131954), and the working solution was obtained by a 1:10 dilution in water to a final concentration of 100 μM, following the protocol described by Artal-Sanz and Tavernarakis [43]. A volume of 40 μL of the working solution was applied to 60 mm × 15 mm plates. Plates were incubated in the dark for 1 h to allow uniform diffusion of the dye, after which L4-stage worms were transferred and incubated for at least 16 h at 20 °C. Following incubation, worms were immobilized on 5% agarose pads using polystyrene nanoparticles (Alpha Aesar, Cat. #42712) as described above. Image acquisition was performed within 30 min using the Nikon A1R confocal microscope with the 60 × /1.4 NA objective and the Deep Red laser channel. Image processing and mitochondrial quantification were conducted using the same workflow described for tissue-specific imaging.

### 2.9 TMRE/mitotracker red CMXRos and green staining and imaging

To assess mitochondrial membrane potential, Tetramethylrhodamine, Ethyl Ester, Perchlorate (TMRE) (Invitrogen, Cat. #T669) dye was diluted to a concentration of 10 mM in DMSO, added to molten NGM medium to a 2.5 μM final concentration and poured into plates, which were then allowed to dry in the dark for 30 min. Plates were subsequently seeded with *E. coli* OP50 and incubated for 48 h. L4-stage worms were then transferred onto the plates and incubated for at least 16 h at 20 °C.

For the quantification of mitochondrial mass and mitochondrial matrix oxidation status, a combination of MitoTracker™ Green FM and MitoTracker™ Red CM-H_2_XRos were used (Invitrogen, Cat. #M7514 and #M7513, respectively). Both dyes were diluted to 1 mM in DMSO and a working solution was prepared in water at 10 μM. Mitotracker dyes were added onto 35 mm NGM plates previously seeded with *E. coli* OP50 to a 100 nM final concentration. After allowing the plates to dry for 30 min, L4-stage worms were transferred and incubated for a minimum of 16 h at 20 °C.

Prior to imaging for all mitochondrial dyes, worms were cleaned by crawling on unseeded NGM plates for 30 min (TMRE) and 60 min (Mitotrackers). Fluorescence (FITC or TRITC), brightfield, and differential interference contrast (DIC) images were acquired using an Olympus BX-61 upright microscope equipped with UPlanFl 10x/0.30 objective (U Plan Fluor series) and DP72 digital camera coupled to CellSens Software for image acquisition and analysis. Animals were mounted on 2% agar pads and anesthetized with 10 mM levamisole prior to imaging. The whole animals were quantified by ImageJ.

### 2.10 MitoSOX red assay

Mitochondrial superoxide anion production was determined using MitoSOX Red™ (Invitrogen, Cat. #M36008). To this purpose, nematodes were synchronized and grown on solid NGM plates until reaching L4 stage, at this point treatment with MitoSOX Red™ was started in liquid culture as previously described [44]. Specifically, nematodes were transferred to liquid media in a 96-well plate, MitoSOX Red™ was added to the wells at a final concentration of 10 μM and the plate incubated in the dark at 20 °C with mild shaking. After 24 h the animals were washed three times with S-Medium and transferred onto new solid, OP50 seeded plates. The worms were incubated on the plates for 20 min to let them clean their guts. For imaging, nematodes were mounted on microscopic slides and anesthetized by adding 10 mM levamisole. Images were acquired immediately with a Zeiss AxioImager M1 microscope (Carl Zeiss Inc., Germany) using a 40x objective and a DsRed filter. Finally, the worm head region, specifically the posterior pharyngeal bulb, was manually selected and the integrated intensity was analyzed with Image J/Fiji 2.3 [45].

### 2.11 Oxygen consumption rate (OCR) measurement

The oxygen consumption rate (OCR) was measured using a Seahorse XFp Analyzer (Agilent Technologies). Approximately 25 *C. elegans* at day 1 adult stage were transferred into each well of Seahorse XFp cell culture miniplates pre-filled with M9 buffer. OCR was recorded at 20 °C, eight times under basal conditions, ten times after carbonyl cyanide 4-(trifluoromethocy)phenylhydrazone (FCCP) injection (for maximal respiration) and four times after sodium azide (for no mitochondrial respiration). Working solutions were prepared in M9 buffer at the following final concentrations: FCCP (Sigma-Aldrich, Cat. #C2920) at 250 μM and sodium azide (Sigma-Aldrich, Cat. #S2002) at 400 mM. Basal OCR was defined as the average of the last five initial measurements. Post-treatment OCR was calculated as the average of the last four measurements after FCCP injection and last two after sodium azide injection. Values were normalized to the number of worms per well. A minimum of three independent experiments were performed.

### 2.12 ATP levels measurement

ATP levels were measured according to the published protocol [46], with minor modifications, as described below. For each assay, approximately 100 day 1 adult worms were used for ATP and total protein extraction. The resulting supernatants were diluted threefold to obtain a final volume of 150 μL. ATP concentrations were determined using a bioluminescence-based ATP Determination Kit PRO, 10 mL (Biaffin GmbH & Co., Cat. #LBR-P010), following the manufacturer's instructions. Luminescence was measured with a CLARIOstar plate reader (BMG LabTech) using Invitrogen™ microplates designed for fluorescence-based assays (Cat. #M33089). ATP standard curves were prepared at concentrations of 5, 10, 15, and 25 μM.

### 2.13 *Staphylococcus aureus* and *Pseudomonas aeruginosa* infection assay

Bacterial infection assays were performed using *Staphylococcus aureus* strain SH1000 *telA::KAN* and *Pseudomonas aeruginosa* strain PA14 following standard methods [47], with minor modifications as detailed next. *S. aureus* was cultured in Tryptic Soy Broth (TSB; BD Biosciences, Cat# 211825) supplemented with 50 μg/ml kanamycin (Sigma-Aldrich, Cat# K1377) overnight (37 °C, 200–220 rpm), and 10 μl was spread on Tryptic Soy Agar plates (TSA; BD Biosciences, Cat# 236950) containing 10 μg/ml kanamycin, incubated at 37 °C for 5 h, and shifted to 25 °C overnight. *P. aeruginosa* was cultured overnight in Luria-Bertani broth (LB; BD Biosciences, Cat# 244620), and 10 μl was spread on Nematode Growth Media (NGM) slow killing plates [prepared using Bacto Agar (BD Biosciences, Cat# 214010) and Bacto Peptone (BD Biosciences, Cat# 211677)], incubated at 37 °C for 24 h, and shifted to 25 °C for 24 h to obtain full bacterial lawns. *C. elegans* were grown to the L4 larval stage on *E. coli* OP50 at 20 °C. For both assays, L4 animals were treated with 100 μg/ml 5-fluoro-2′-deoxyuridine (FUDR; Sigma-Aldrich, Cat# F0503) on solid media overnight at 15 °C. Animals were subsequently transferred to infection plates lacking FUDR. Three plates each containing 30–40 animals were examined for each genotype. Infection assays were carried out at 25 °C. Survival was quantified using standard methods [47]; animals dying of disrupted vulva or crawling on walls were censored for analysis.

### 2.14 Paraquat and juglone tolerance assay

Juglone (Sigma-Aldrich, Cat. #H47003) and paraquat (Sigma-Aldrich, Cat. # 856177) were dissolved in DMSO and Milli-Q water and then added to molten NGM to obtain final concentrations of 150 μM (juglone) and 50 mM (paraquat). The media were thoroughly mixed and poured onto 60 mm (juglone) or 35 mm (paraquat) diameter plates. Once solidified, plates were seeded with 50 μL of *E. coli* OP50 and allowed to dry before use. L4-stage worms were then transferred and incubated at 20 °C for 16 h before scoring for live or dead animals. Worms were considered alive if they responded to gentle touch with a platinum pick on the agar surface or on the head or showed pharyngeal pumping. Worms desiccated on the wall of the plate were censored and excluded from the analysis.

### 2.15 Rotenone and antimycin a tolerance assay

Rotenone (Sigma-Aldrich, Cat. #R8875) and antimycin A (Sigma-Aldrich, Cat. #A8674) mitochondrial electron transport chain (ETC) inhibitors, were dissolved in DMSO to a stock solution of 5 mg/ml and 10 mg/ml and then added to molten NGM medium to final concentrations of 1 μM and 20 μM, respectively. Once solidified, plates were seeded with 50 μL of *E. coli* OP50 and allowed to dry before use. L4-stage worms were then transferred and incubated at 20 °C for 16 h before scoring for live or dead animals. Worms were considered alive if they responded to gentle touch with a platinum pick on the agar surface or on the head or showed pharyngeal pumping. Worms desiccated on the wall of the plate were censored and excluded from the analysis.

### 2.16 Glutathione redox status determination

To assess GSH/GSSG ratio we used transgenic strains expressing Grx1-roGFP2 in the cytoplasm from the *rpl-17* ubiquitous promoter [48] or in the mitochondrial matrix of muscle cells from the *myo-3* promoter [49]. Approximately 250 synchronized day-1 adult worms were transferred using a glass pipette into 96-well fluorescence-based assay microplates (Invitrogen, Cat. #M33089) and resuspended in 80 μL of M9 buffer. At least three biological replicates were analyzed, with at least three technical replicates (wells). Fluorescence measurements were performed using a CLARIOstar microplate reader (BMG LabTech). Fluorescence intensity (FI) was recorded in multichromatic mode using endpoint measurements with five flashes per well. Two fluorescence channels were acquired per well. The first channel used an excitation wavelength of 405 ± 15 nm, a 508.2 nm dichroic filter, and an emission wavelength of 525 ± 8 nm. The second channel used an excitation wavelength of 488 ± 15 nm, a 505.2 nm dichroic filter, and an emission wavelength of 525 ± 8 nm. Gain values and focal height were based on previous adjustment procedures, using the entire plate for gain calibration and a specific well for focal height determination. A settling time of 0.1 s was applied prior to reading. Plates were read bidirectionally, horizontally from left to right and from top to bottom. Temperature control, as well as O_2_ and CO_2_ regulation, were disabled during measurements, and no injection system was used.

Data were acquired using the SMARTcontrol software and analyzed by the MARS software data analysis. The mean fluorescence of blank wells corresponding to each biological replicate (plate) was subtracted from the fluorescence values of wells containing worms at each excitation wavelength. Corrected fluorescence values were then used to calculate excitation ratios. For strains expressing Grx1-roGFP2 biosensor, the redox status is reported as the oxidized-to-reduced ratio, calculated as fluorescence excited at 488 nm divided by fluorescence excited at 405 nm.

### 2.17 Lifespan assays

Lifespan analyses were carried out with standard procedure in the field [50]. Briefly, age-synchronized population of 60-80 worms were used to start survival analysis. To avoid cross generation contamination worms were transferred to fresh plates every day during the fertile phase while every other day from the end of the fertile period. Animals not able to move upon pick-prodding and with no pharyngeal pumping were scored as dead. Survival analysis was performed with OASIS 2 [51] using the Kaplan Meier estimator. Statistical differences were evaluated using the log-rank test between the pooled population of worms and p-values were adjusted for multiple comparisons by Bonferroni method.

### 2.18 Embryonic and larval lethality assay

To quantify embryonic and larval lethality, 15–20 synchronized hermaphrodites were allowed to lay eggs for 2-3 h and laid embryos were determined. After three days of incubation at 20 °C the developmental stages of the progeny were quantified in a stereomicroscope.

### 2.19 CeleST swimming activity assay

Swimming behavior was assessed at day 1 of adulthood. A glass slide with a 10 mm pre-printed ring was prepared and loaded with 50 μl M9 Buffer. Five worms were placed in the swimming area, for a total of 45 animals per condition. Worms were allowed to settle for 20s before starting the recording. 30s videos with at 16 frames per second of the animals were taken using a Nikon LV-TV microscope with a OPTIKA C–P20CM camera and processed by CeleST software [52].

### 2.20 Pharyngeal pumping

Pharyngeal pumping measurements were performed by recording 20-s videos for each first day adult worm using a Leica MDG36 fluorescence stereo microscope equipped with a DMK 31AU03 monochrome camera. Video acquisition was carried out with IC Capture 2.3 software. Pharyngeal pumps were manually counted using video playback at 0.42 × speed. Final pumping rates were converted to units of pumps per minute through appropriate time normalization.

### 2.21 Electropharyngeogram

To perform electrical recordings of pharyngeal pumping (electropharyngeograms, or EPGs), we placed the Nemametrix Screen Chip System on an inverted Zeiss Axiovert 200 microscope equipped with an LD A-Plan 20x objective. To minimize interfering electrical noise during the recordings, we used a system grounding shield. Baseline noise was typically between 10 and 40 μV. The experiments were performed at 20 °C. For each experiment, 100 worms at day 4 of adulthood were picked from the culture plate and washed in 1.5 ml of 0.2 μm filtered M9 buffer containing 0.1% Tween. The worms were then washed four times with 0.2 μm filtered M9 buffer, once with M9 buffer containing 2.3 mM serotonin (Aesar Chemicals, Cat. #B21263), and finally suspended in 1 ml of M9 buffer containing 2.3 mM serotonin. They were then left to settle for 15 min. All experiments were performed within 15–90 min of initial serotonin exposure. We loaded a NemaMetrix Screen Chip System (NemaMetrix, Eugene, OR, USA; Cat. #SK100) with a fresh SC40 Screen Chip (NemaMetrix, Eugene, OR, USA; Cat. #SKU0002) and added an M9 buffer solution containing 2.3 mM serotonin. After initiating the NemAcquire software and recording the basal power line noise, the worms were vacuum-loaded onto the SC40 Screen Chip to start the experiment. The 1 Hz high-pass and 50 Hz notch filter settings were selected. An EPG recording lasting 180 s was made for each animal, and records from 20 to 25 animals were obtained for each replicate. These records were analyzed using NemAnalysis v0.2 software. Experiments with a frequency of less than 0.1 Hz or a pump duration coefficient of variation greater than 50% were censored.

### 2.22 Mitochondrial calcium imaging

Measurements of mitochondrial Ca^2+^ in the pharynx were performed in worms expressing the *Ex [Pmyo-2::2mt8::YC3.60]* reporter [53] worms at day 5 of adult life. The worms were glued onto an agar pad (2% agar in M9 buffer) on a coverslip using Dermabond Topical Skin Adhesive (Johnson & Johnson), and 2.3 mM serotonin (Aesar Chemicals, Cat. #B21263) was added to stimulate pumping. The coverslip containing the glued worm was mounted in a chamber on the stage of a Zeiss Axiovert 200 inverted microscope. Fluorescence was excited at 430 nm using a Cairn monochromator with a 15 nm bandwidth, and images of the emitted fluorescence were obtained using a Zeiss C-Apochromat 40x/1.2 W objective. These images were collected using a 450 nm long-pass dichroic mirror and a Cairn Optosplit II emission image splitter, which produced separate emission images at 480 nm and 535 nm. The splitter contained DC/ET480/40 m and DC/ET535/30 m emission filters and a FF509-FDi01-25x36 dichroic mirror (all from Chroma Technology). A Hamamatsu ORCA-ER camera recorded simultaneous 200 ms images at the two emission wavelengths continuously at a rate of 2.5 Hz. Both images were then ratioed to obtain 535 nm/480 nm fluorescence ratio images of the pharynx terminal bulb. Experiments were performed at 20 °C, with basal fluorescence being recorded for 15 min prior to the addition of carbachol to a final concentration of 10 mM to induce maximum mitochondrial Ca^2+^ uptake.

Fluorescence was recorded and analyzed using the Metafluor programme (Universal Imaging). The traces shown represent either the 535 nm or 480 nm emission fluorescence, or the 535 nm/480 nm fluorescence emission ratio, of the pharynx terminal bulb. [Ca^2+^] peaks in the ratio were only considered acceptable when inverted changes at both wavelengths were clearly observed, as shown in Fig. 6e and f. The fluorescence intensities and changes in the ratio were analyzed using an algorithm designed to calculate the width at baseline and mid-height, both of which were expressed in seconds. The height was obtained as a percentage of the change in the ratio, and the frequency was measured at each peak by dividing 9 by the time interval including that peak and the four peaks before and after it. The mean value was calculated as an average of all the individual values obtained for each parameter.

### 2.23 Hypodermal wounding

We wounded the epidermis of young adult stage animals using a Micropoint UV laser or needle. All images before and after wounding were taken using a spinning disk confocal microscope (Andor 100x, NA 1.46 objective). Actin ring quantitation and survival rate were performed as previously described [54]. The nuclear intensity analyses were performed in ImageJ. Background intensity was used as a threshold. The intensity was analyzed using Analyze Particles (a function in ImageJ software) automatically with minimum size at 0.2 square micrometers. The value of intensity was automatically calculated and analyzed using GraphPad. At least 20 wounded young adult animals were examined. For cluster number, 600 × 400-pixel around the wounded area was chosen for analysis.

### 2.24 Fertilization capacity

To assess male fertility, *fem-1(hc17)* hermaphrodites (grown at 25 °C) were crossed with wild-type or *gsr-1a trxr-2* young adult males in a 4:1 ratio. After 24 h, males were removed and hermaphrodites were transferred to a new plate at 20 °C. The hermaphrodites were transferred daily until they stopped laying eggs. The total brood size was counted. Hermaphrodites that died or were absent before the end of the experiment were excluded from the analyses [55].

### 2.25 Exophers

Exopher quantification followed the procedures outlined by Turek et al. [10,56]. Briefly, exophers were assessed in animals expressing the *wacIs14[Pmyo-3::tomm-20_1-50aa::attB5::mgfp; unc-119(+)]* transgene and imaged in a Zeiss Axio Zoom. V16 stereomicroscope equipped with 63 HE and 38 HE filter sets. Worms were age-matched by manually selecting 3-fold stage embryos under a stereomicroscope. On the second day of adulthood, animals were examined directly on NGM plates, and the number of exophers produced by each freely moving worm was counted**.**

### 2.26 Graphical and statistical analysis

Data were processed in Excel (Microsoft Corporation) then Prism (GraphPad Software) was used to generate curve and bar charts and perform the statistical analyses described in the Figure Legends.

## 3 Results

### 3.1 Inactivation of mitochondrial glutathione reductase and thioredoxin reductase causes germline defects and small body size

GSH is the main redox currency in the cell and its key role in mitochondrial function is illustrated by the inability to proliferate of mammalian cells lacking GSH mitochondrial importers SLC25A39 and SLC25A40 [57]. This essential role of GSH in mitochondria is conserved in *C. elegans* as a null allele of the only worm SLC25A39/SLC25A40 orthologue *C16C10.1(syb5556)* results in a fully penetrant L1 larval lethality and can only be propagated as a balanced heterozygous (strain VZ1095, Supplementary Table 1). Once inside mitochondria, GSH redox homeostasis is maintained by the redundant action of the mitochondrial thioredoxin and glutathione/glutaredoxin systems in organisms as distant as yeast or *Arabidopsis thaliana* (Gostimskaya and Grant 2016; [29]).

**Fig. 1:**
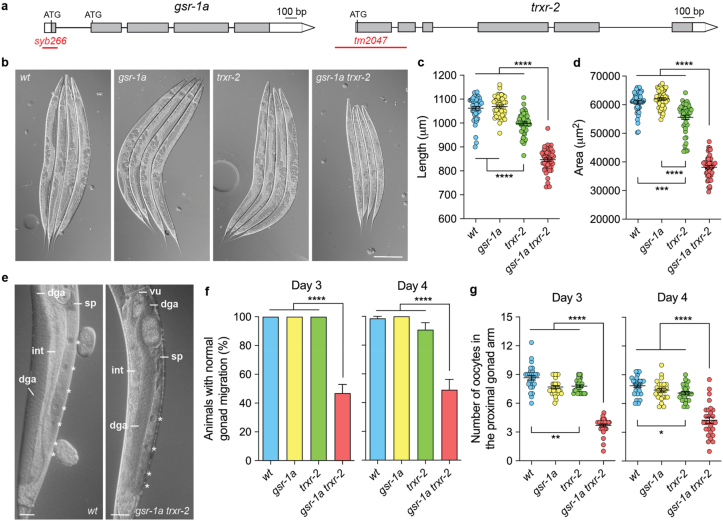
**Genomic organization of *gsr-1* and *trxr-2* genes and visible phenotypes of *gsr-1a(syb266)* and *trxr-2(tm2047)* mutants.** a) Boxes represent exons and lines show spliced introns. White boxes indicate 5′-UTR and 3′-UTR, respectively, and grey boxes indicate the ORF. Boundaries of *gsr-1a* and *trxr-2* deletions are shown as red lines. b) Representative micrographs of animals of the specified genotypes at third day after synchronized egg-lay. Scale bar 200 μm. Quantification of c) worm length and d) worm area. Data are from two independent experiments with at least 25 animals per assay. Error bars are SEM. ∗∗∗p < 0.001; ∗∗∗∗p < 0.0001 by Kruskal-Wallis test. e) Representative micrographs of wild-type controls and *gsr-1a trxr-2* double mutants at third day after synchronized egg-lay. dga, distal gonad arm; int, intestine; sp, spermatheca; vu, vulva. Scale bar 25 μm. f) Quantification of worms with gonad migration defects. Data are the mean ± SEM from three different experiments with at least 20 animals per assay. ∗∗∗∗p < 0.0001 by Kruskal-Wallis test. g) Quantification of the oocytes in the proximal gonad arm. Data are from three different experiments with at least 20 animals per assay. Error bars are SEM. ∗p < 0.05; ∗∗p < 0.01; ∗∗∗∗p < 0.0001 by Kruskal-Wallis test.

To test whether this functional redundancy is also present in metazoan, we investigated the phenotypic consequences of disrupting mitochondrial redox homeostasis in *C. elegans* by combining the previously characterized *trxr-2(tm2047)* null allele of mitochondrial thioredoxin reductase [58] (Fig. 1a) with a newly generated CRISPR-Cas9 *gsr-1(syb266)* deletion allele, which removes the first exon of *gsr-1* (Fig. 1a). This exon encodes the mitochondrial targeting sequence of GSR-1a isoform, so the *gsr-1(syb266)* allele permits expression of the essential cytosolic isoform GSR-1b, while eliminating expression of the mitochondrial isoform GSR-1a [18].

While the single mutants appeared superficially wild type with no overt phenotype, the *gsr-1a trxr-2* double mutants displayed a significantly smaller size, which was already evident at the L4 stage (two days at 20 °C after synchronized egg-lay) (Sup Fig. 1), phenotype that became more pronounced by the first day of adulthood (Fig. 1b–d). Additionally, we observed a high incidence of gonad migration defects, characterized by thinning and mislocalization of the distal gonad arm relative to the intestine, sometimes extending into the vulva area (Fig. 1e and f). There was also reduced thickness of the proximal gonad arm, which contained fewer and slimmer oocytes, along with a distorted spermatheca (Fig. 1e–g).

### 3.2 *gsr-1a trxr-2* double mutants exhibit normal developmental timing but have reduced brood size and extended egg-lay period

Interestingly, during the isolation of the *gsr-1a trxr-2* double mutants, we observed that OP50 plates seeded with double mutant candidates became saturated noticeable later than those seeded with either single mutants or wild-type controls, suggesting a developmental delay and/or reduced progeny production in the double mutants, both phenotypes previously associated with mitochondrial dysfunction [[59], [60], [61]].

**Fig. 2:**
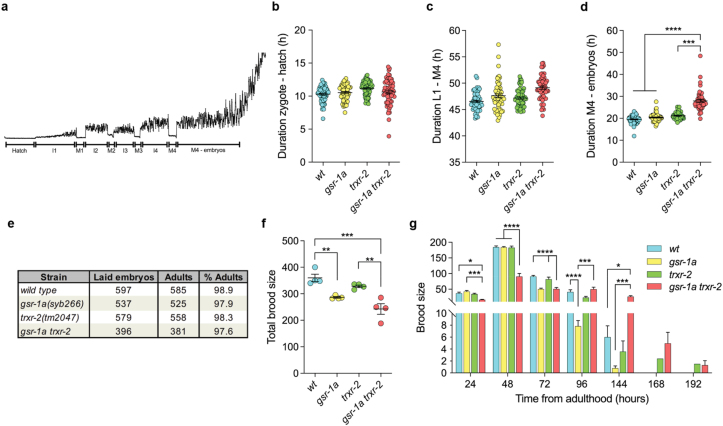
**Developmental time and progeny phenotypes of *gsr-1a(syb266)* and *trxr-2(tm2047)* mutants.** a) Representative bioluminescence profile of a wild-type worm from the zygote stage until the egg-lay period initiation (distorted signal). I1–I4 intermolt intervals, M1-M4 molt intervals, devoid of signal due to halted food ingestion. Quantitative analysis of b) ex-uterus embryonic development, c) larval development, d) egg-lay initiation period. Data are from three different experiments with three biological replicates per assay. Error bars are SEM. ∗∗∗p < 0.001; ∗∗∗∗p < 0.0001 by ordinary One-way ANOVA (b,c) and by Kruskal-Wallis test (d). e) Percentage of animals that reach adulthood. Data are pooled from two independent experiments with three biological replicates. f) Quantification of the total brood size of 20 animals. Each point represents the average progeny of 5 animals on the same plate. Error bars are SEM. ∗∗p < 0.01; ∗∗∗p < 0.001 by ordinary One-way ANOVA. g) Bars show the progeny per day from 20 animals of each genotype. Data are the mean ± SEM. ∗p < 0.05; ∗∗∗p < 0.001; ∗∗∗∗p < 0.0001 by ordinary One-way ANOVA.

To directly assess whether the reduced plate saturation reflected a bona fide developmental delay, we quantified the duration of embryonic and larval stages in the double mutants using the luciferase-based method developed by Olmedo et al. [33] that accurately quantifies the duration of all molt and intermolt periods as well as the time from egg-lay to hatching and from the last molting step until the lay of the first embryos (Fig. 2a). We found that the embryonic and larval development of *gsr-1a trxr-2* animals do not differ from that of wild type and *gsr-1a* or *trxr-2* single mutant controls (Fig. 2b and c). However, we noticed that the period from the last molt until the first embryos are laid was significantly longer in *gsr-1a trxr-2* double mutants (Fig. 2d) suggesting problems with progeny production caused by oocyte production, sperm depletion or defective mitochondrial signaling in the germline. To test this, we first ruled out the occurrence of embryonic or larval lethality in *gsr-1a trxr-2* double mutants (Fig. 2e). Next, we measured total brood size and found that *gsr-1a* mutants had reduced progeny as compared to that of wild-type or *trxr-2* mutant controls while *gsr-1a trxr-2* double mutants had the lowest brood size, although it did not differ significantly from that of *gsr-1a* single mutants (Fig. 2f). Finally, due to the delayed time from last molt to egg-lay of *gsr-1a trxr-2* double mutants, we measured the interval during which the four strains produce progeny and found that double mutants have an extended egg-lay period (Fig. 2g). Thus, *gsr-1a trxr-2* double mutants show normal somatic development but exhibit delayed reproductive maturation, reduced progeny output and an extended egg-lay window, revealing a specific sensitivity of reproductive physiology associated to mitochondrial redox defects.

### 3.3 Disruption of mitochondrial redox homeostasis induces mitochondrial UPR

**Fig. 3:**
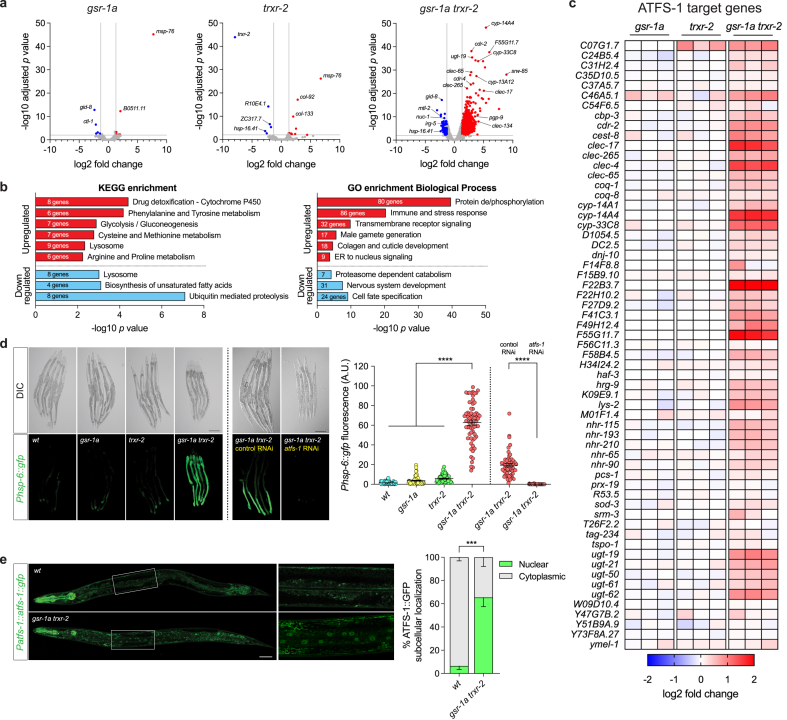
**Transcriptomic analysis of *gsr-1a(syb266)* and *trxr-2(tm2047)* mutants.** a) Volcano-plots of *gsr-1a* and *trxr-2* single and double mutants. The Log2 fold change indicates the mean expression levels for each gene, represented by dots). No differentially expressed genes (DEG) are indicated in grey, upregulated genes in red and downregulated genes in blue. Data are from three independent experiments with three biological replicates analyzed with the edgeR package [37]. b) Gene Ontology and KEGG pathway enrichment clustering of DEGs (FC > 2, p < 0.05) between *gsr-1a trxr-2* double mutants and wild-type controls. c) Heat-map of the high confidence ATFS-1 target genes [62]. The color-coded heat map represents gene expression differences (log2 fold change) relative to the levels found in wild-type controls. d) Representative differential interference contrast and fluorescence micrographs and quantification of worms at first day of adulthood expressing GFP under the control of the *hsp-6* promoter. Data are from three different experiments with at least 20 animals per assay. Error bars are SEM. ∗∗∗∗p < 0.0001 by Kruskal-Wallis test. Scale bar 200 μm. e) Representative fluorescence micrographs and quantification of L4 worms expressing ATFS-1:GFP under the control of the *atfs-1* promoter. Data are the mean ± SEM of three independent experiments with at least 70 animals per genotype. ∗∗∗p < 0.001 by two-tailed Mann-Whitney test. Scale bar 50 μm.

To gain deeper insight at the molecular level on the consequences of disrupting redox homeostasis in mitochondria, we performed a RNAseq analysis. Consistent with the lack of phenotype in *gsr-1a* and *trxr-2* single mutants, we only observed a minor alteration in the mRNA expression pattern of *gsr-1a* or *trxr-2* animals (Fig. 3a). In contrast, *gsr-1a trxr-2* double mutants showed a dramatic alteration of their transcriptional profile with hundreds of genes up and downregulated, some of which were validated by qPCR (Fig. 3a, Sup Fig. 2a and b and Supplementary Tables 2 and 3). To identify the biological processes and the biochemical pathways in which these genes participate we performed a Gene Ontology and KEGG enrichment analysis [63,64] and found a strong upregulation of genes related to protein phosphorylation, detoxification and immune stress response, transmembrane receptor signaling, male gamete generation, metabolism of sulfur containing amino acids and cuticle development. In turn, downregulated genes were mainly involved in proteasomal function and cell specification processes (Fig. 3b).

The induction of genes involved in drug detoxification, pathogen infection and mitochondrial repair is a common response to mitochondrial dysfunction as a result of disrupting core cellular functions [65,66]. Interestingly, among the most upregulated genes in the *gsr-1a trxr-2* double mutant we found many high confidence ATFS-1 target genes [62] (Fig. 3c), suggesting that these animals may have an induced UPR^mt^. To test this, we used the UPR^mt^ reporter transgene *zcIs13[Phsp-6::gfp]* [67] and found that only the *gsr-1a trxr-2* double mutant but not the respective single mutant controls strongly induced GFP expression (Fig. 3d). Importantly, this response was fully dependent on ATFS-1 transcription factor as *atfs-1* RNAi downregulation completely abolished the fluorescence in *gsr-1a trxr-2; zcIs13[Phsp-6::gfp]* animals (Fig. 3d). Consistently, *gsr-1a trxr-2* double mutants have a constitutive nuclear localization of ATFS-1, prerequisite to trigger a UPR^mt^ transcriptional response (Fig. 3e) [4]. The relevance of ATFS-1 function in the survival of *gsr-1a trxr-2* worms is highlighted by the fact that a triple mutant *gsr-1a trxr-2; atfs-1* is not viable and can only be maintained as a balanced strain (strain VZ1063, Supplementary Table 1). Moreover, while canonical ATFS-1 dependent UPR^mt^ is strongly induced in *gsr-1a trxr-2* double mutants (approx. 50-fold induction) (Fig. 3d), other mitochondrial related stress responses such as the ATFS-1 independent UPR^mt^ [68], mitochondrial to cytosolic stress response (MCSR) [69] or ethanol and stress response element (ESRE) [70], as well as UPR^er^ [71] were only very marginally altered (Sup Fig. 2c).

Collectively, the phenotypic and transcriptomic analyses show that GSR-1a and TRXR-2 are functionally redundant in mitochondria and that their simultaneous absence induces a robust ATFS-1 dependent UPR^mt^ response that is essential for the survival of animals with compromised redox homeostasis in this organelle.

### 3.4 Induction of mitochondrial UPR does not enhance stress resistance or increase lifespan in *gsr-1a trxr-2* mutants

*C. elegans* continuously monitors the correct function of core cellular activities such as protein synthesis, proteasome-dependent degradation or mitochondrial respiration. Disruption of these essential cellular activities is perceived as an external insult, triggering a defensive transcriptional program that includes genes involved in pathogen resistance, xenobiotic detoxification and innate immune response [65]. The transcriptomic profile of *gsr-1a trxr-2* mutants, which is enriched for genes within these pathways (Fig. 3b), resembles the transcriptional responses of mutants compromising core mitochondrial functions such as the electron transport chain, tricarboxylic acid cycle or iron-sulfur cluster biogenesis [65,66,72,73]. These similarities suggest that maintenance of mitochondrial redox homeostasis is among the key mitochondrial functions under cellular surveillance that are essential for organismal survival.

**Fig. 4:**
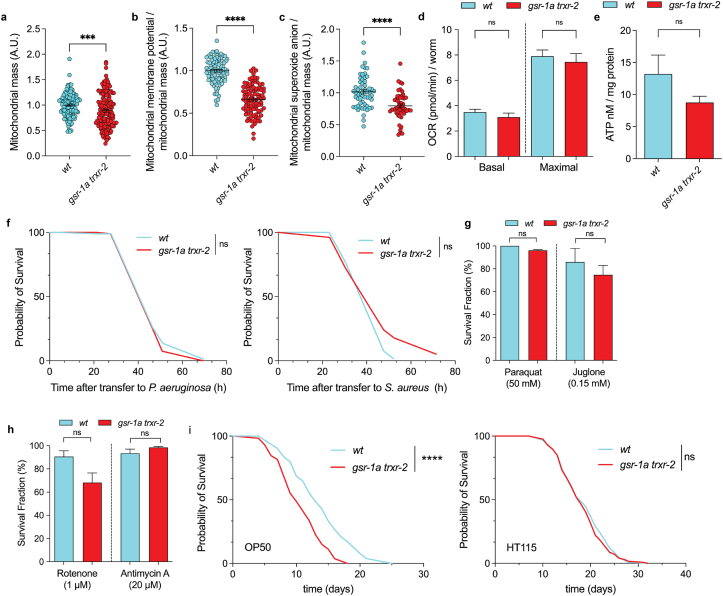
**Mitochondrial function, stress response, and lifespan in *gsr-1a(syb266) trxr-2(tm2047)* mutants.** a) Quantification of mitochondrial mass using MitoTracker™ Green, b) mitochondrial membrane potential using TMRE and c) mitochondrial superoxide anion production using mitoSOX Red™. All measurements were performed on first day adults and data are from three different experiments with at least 40 animals per assay. ns, not significant; ∗∗∗p < 0.001 and ∗∗∗∗p < 0.0001 by two-tailed Mann–Whitney test. Error bars are SEM. d) Basal/maximal oxygen consumption rate normalized to the number of worms and e) ATP content normalized by total protein amount. Data are the mean ± SEM from three different experiments with at least 50 animals per assay. ns, not significant by ordinary One-way ANOVA (d) or two-tailed Mann–Whitney test (e). f) Survival curves following exposure to *Pseudomonas aeruginosa* (left) and *Staphylococcus aureus* (right). Data are from three independent assays for *P. aeruginosa* and two for *S. aureus*, respectively. ns, not significant by Log-rank (Mantel–Cox) test. g) Quantification of survival after 16 h exposure to paraquat and juglone and h) to rotenone and antimycin. All measurements were performed on L4 animals and data are the mean ± SEM from three different experiments with at least 40 animals per assay. ns, not significant and ∗p < 0.05 by two-tailed Mann–Whitney test. i) Survival curves of animals fed on *E. coli* OP50 (left) or HT115 (right) bacteria. Data are from three independent assays on OP50 and two on HT115. ns, not significant and ∗∗∗∗p < 0.0001 by Log-rank (Mantel–Cox) test with Bonferroni correction.

Because robust transcriptional remodeling and UPR^mt^ activation were only found in the *gsr-1a trxr-2* double mutant, but not in the respective single mutants, subsequent analyses focused only on the double mutant. We first assessed mitochondrial content and found it slightly decreased in the *gsr-1a trxr-2* worms compared to wild-type controls (Fig. 4a). Notably, mitochondrial membrane potential and superoxide production were also significantly reduced in the *gsr-1a trxr-2* mutant (Fig. 4b and c) whereas respiration and ATP levels remained unchanged (Fig. 4d and e). Despite the transcriptional activation of stress and defense-related genes, *gsr-1a trxr-2* animals did not exhibit enhanced resistance to the bacterial pathogens *Pseudomonas aeruginosa* or *Staphylococcus aureus* (Fig. 4f and Supplementary Table 4), nor to mitochondrial toxicants such as paraquat and juglone (Fig. 4g) or the mitochondrial complex I and III inhibitors rotenone and antimycin A, respectively (Fig. 4h). Finally, we also measured glutathione redox status in the cytosol of whole worms [48] and in mitochondria of muscle cells [49] and found no difference between the double mutant and wild-type control animals (Sup Fig. 3a and b). This is consistent with no changes in whole-animal mitochondrial matrix oxidation levels measured with MitoTracker™ Red CM-H_2_XRos probe (Sup Fig. 3c).

The relationship between UPR^mt^ induction, typically through depletion of mitochondrial genes, and lifespan modulation is well established, with outcomes depending on the degree of mitochondrial perturbation [[74], [75], [76]]. Worms with impaired mitochondrial function often display reduced body size and reduced progeny yet increased lifespan (reviewed in Ref. [77]). We therefore tested whether disruption of mitochondrial redox homeostasis also affects longevity. Unexpectedly, we found that *gsr-1a trxr-2* worms are short lived compared to wild-type animals when grown on OP50 bacteria, whereas no differences were detected on HT115 bacteria (Fig. 4i and Supplementary Table 5). These results suggest that the impact of impairing mitochondrial redox homeostasis on lifespan may be modulated by nutritional cues.

Taken together, these findings indicate that loss of mitochondrial redox homeostasis does not markedly affect stress resistance or mitochondrial physiology in *C. elegans*, likely due to compensatory activation of UPR^mt^-dependent protective pathways, and consistent with the lethality of the *gsr-1a trxr-2; atfs-1* triple mutant.

### 3.5 Impairment of mitochondrial redox homeostasis alters mitochondrial dynamics without disturbing mitophagy

The observation that *gsr-1a trxr-2* animals are not sensitized to stress despite activation of UPR^mt^ prompted us to investigate whether changes in mitochondrial dynamics, which can be regulated by redox mechanisms [78], underlie the preservation of mitochondrial function in these mutants. To this end, we focused on muscle and hypodermal cells, two tissues with high metabolic activity and energy demand, as muscle contractility is required for animal continuous movement, whereas the hypodermis plays a central role in stress resistance, coordinating systemic responses with other tissues through the release of metabolic and hormonal signals [[79], [80], [81]].

**Fig. 5:**
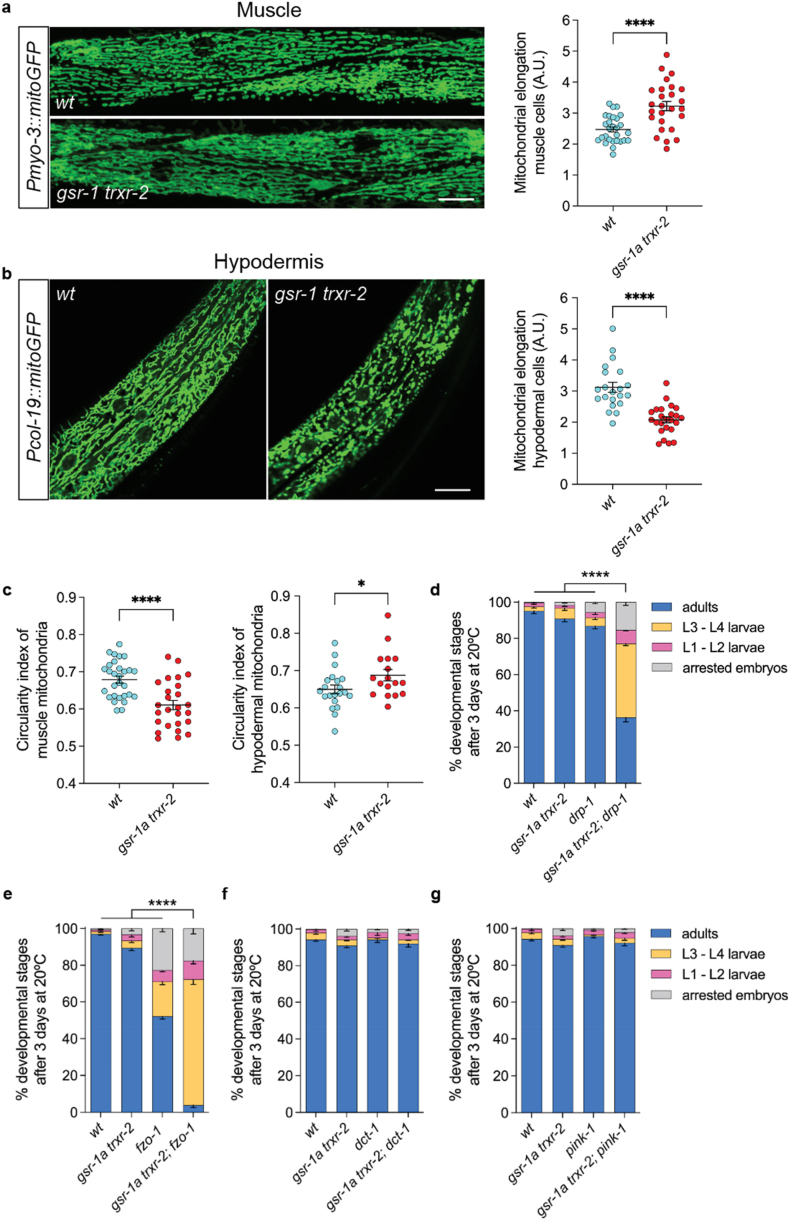
**Mitochondrial tissue-specific dynamics of *gsr-1a(syb266) trxr-2(tm2047)* mutants.** Representative fluorescence micrographs of mitochondrial network in a) body wall muscle cells and b) hypodermal cells. Graphs show the quantification of mitochondrial elongation from at least 20 first day adults for both tissues. ∗∗∗∗p < 0.0001 by unpaired *t*-test. Error bars are SEM. Scale bar 20 μm. c) Quantification of the circularity index of mitochondrial from muscle (left) and hypodermal (right) cells. Data are from at least 20 first day adults. ∗p < 0.05; ∗∗∗∗p < 0.0001 by unpaired *t*-test. Error bars are SEM. d-g) Percentage of developmental stages in worms with defective mitochondrial fission-fusion and mitophagy. Data are the mean ± SEM from three different experiments with at least 100 animals per assay. ∗∗∗∗p < 0.0001 by ordinary One-way ANOVA.

Interestingly, *gsr-1a trxr-2* double mutants exhibited tissue-specific alterations in mitochondrial morphology with muscle cells having more elongated mitochondria as compared to wild-type controls, whereas those in hypodermal cells displayed increased fragmentation (Fig. 5a and b). This differential morphology was confirmed by quantifying mitochondrial circularity, a widely used parameter for assessing mitochondrial network integrity (Fig. 5c) [82]. Mitochondrial dynamics are governed by the balance between mitochondrial biogenesis and degradation (mitophagy), as well as by the rates of fusion and fission events, processes modulated by the UPR^mt^ [[83], [84], [85]]. In this context, disruption of mitochondrial fission using *drp-1* mutants or mitochondrial fusion with *fzo-1* mutants severely compromised the development of *gsr-1a trxr-2* double mutants (Fig. 5d and e) whereas inhibition of mitophagy through *dct-1* or *pink-1* mutations had no effect (Fig. 5f and g). Indeed, mitophagy is only slightly increased in *gsr-1a trxr-2* double mutants as shown by the ratio between pH sensitive GFP (autophagosome) and pH-insensitive DsRed (autolysosome) markers of the mtRosella reporter (Sup Fig. 3d) [38].

**Fig. 6:**
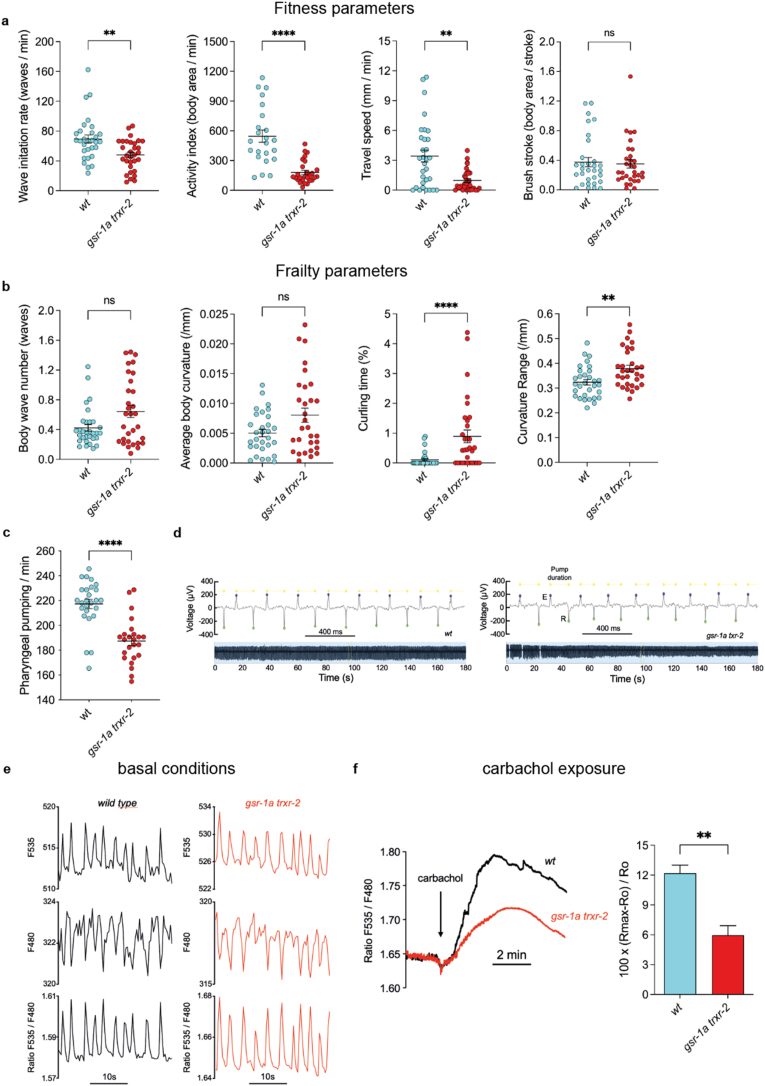
**Muscle-specific phenotypes in *gsr-1a(syb266) trxr-2(tm2047)* mutants.** CeleST locomotory parameters associated with a) physiological fitness and b) physiological frailty in first day adult animals. Data are from three independent experiments with at least 10 animals per assay. ns, not significant; ∗∗p < 0.01; ∗∗∗∗p < 0.0001 by two-tailed Mann-Whitney test. Error bars are SEM. c) Quantification of pharyngeal pumping rate per minute. Data are from three independent experiments with at least 10 animals per assay. ∗∗∗∗p < 0.0001 by two-tailed Mann-Whitney test. Error bars are SEM. d) Representative electropharyngeograms (EPGs) measurements performed in animals at fourth day of adulthood. Measurements were performed on 31 wild-type worms and 30 *gsr-1a trxr-2* double mutants. The following parameters were obtained and pooled: frequency, pump duration, amplitude (voltage change from the E wave peak to the R wave peak) and the R/E ratio. The mean data for these parameters are shown in Sup Fig. 4a e) Ca^2+^ oscillations under resting conditions in pharyngeal muscle cells. The figure shows typical records of the two individual F535 and F480 fluorescences (in arbitrary units), and the F535/F480 ratio, which directly reflects changes in Ca^2+^ concentration. Measurements were performed on 24 wild-type worms and 32 *gsr-1a trxr-2* double mutants. The following parameters were obtained and pooled: frequency, width at baseline, width at half-height and height. The mean data for these parameters are shown in Sup Fig. 4b f) Effect of carbachol on mitochondrial Ca^2+^ uptake. The figure shows the average of 17 experiments performed on wild-type worms and 20 experiments performed on *gsr-1a trxr-2* double mutants. Carbachol was added to the chamber when indicated to reach a final concentration of 10 mM. The bar diagram illustrates the height of the Ca^2+^ peak induced by carbachol in both conditions. ∗∗p < 0.001 by unpaired *t*-test.

Together, these findings indicate that mitochondrial network dynamics is tightly regulated in a tissue-specific manner upon disruption of mitochondrial redox homeostasis, even within tissues that are anatomically associated and functionally coordinated such as muscle and hypodermis [86]. Moreover, the dispensability of mitophagy for *gsr-1a trxr-2* double mutants development supports the notion that overall mitochondrial function remains largely preserved, precluding the need for selective degradation of damaged mitochondria, or alternatively, that compensatory mechanisms such as exopher-mediated mitochondria extrusion [9] contribute to the maintenance of a healthy mitochondrial network.

### 3.6 Tissue-specific somatic and germline effects upon impairment of mitochondrial redox homeostasis

Given the differential effects of the *gsr-1a trxr-2* deficiency on mitochondrial dynamics in muscle and hypodermal, we next focused on phenotypes specifically associated to these two tissues. Using the CeleST software, which quantifies locomotory behaviour in liquid medium as a proxy for muscle function [52], we observed that first day adult *gsr-1a trxr-2* animals exhibit reduced locomotor fitness parameters together with an increase in muscle frailty indicators (Fig. 6a and b). These findings suggest a functional decline of muscle cells when mitochondrial redox homeostasis is compromised. Consistent with this interpretation, *gsr-1a trxr-2* double mutants also display a reduced pharyngeal pumping rate (Fig. 6c), a defect that is not attributable to altered muscle depolarization capacity by defective neuronal signaling, as demonstrated by electropharyngeogram measurements (Fig. 6d and Sup Fig. 4a).

Proper regulation of mitochondrial calcium uptake is essential for muscle performance, both to sustain ATP production during contraction and to maintain cytosolic calcium homeostasis [87,88]. Using a mitochondrial-targeted fluorescence reporter expressed in pharyngeal muscle cells [53], we found that *gsr-1a trxr-2* animals show normal basal mitochondrial calcium import compared to wild-type controls (Fig. 6e and Sup Fig. 4b). However, when worms are exposed to carbachol, a chemical that induces a massive calcium entry into the cytoplasm by activation of the nicotinic acetylcholine receptors in the pharyngeal muscle cells [89], we observed a marked reduction in mitochondrial calcium uptake in the *gsr-1a trxr-2* double mutants (Fig. 6f). Together, these findings indicate that mitochondrial redox homeostasis is required to efficiently buffering high levels of calcium in the cytoplasm.

**Fig. 7:**
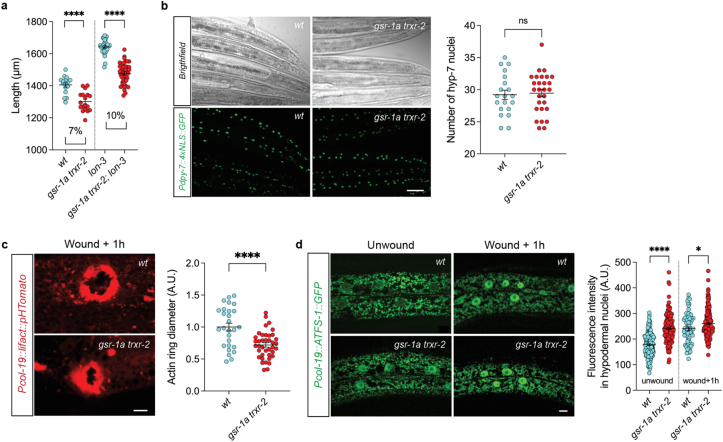
**Hypodermal-dependent phenotypes of *gsr-1a(syb266) trxr-2(tm2047)* mutants.** a) Quantification of day 2 adult animals length. Percentages represent the differential length between indicated genotypes. Data are from three independent experiments with at least 15 animals per assay. ∗∗∗∗p < 0.0001 by two-tailed Mann-Whitney test. Error bars are SEM. b) Representative bright-field and fluorescence micrographs (left) and quantification of syncytium nuclei (right) of the posterior part of hyp-7 cell. Data are from at least 20 animals. ns, not significant by unpaired *t*-test. Error bars are SEM. Scale bar 25 μm. c) Representative fluorescence micrographs (left) and quantification of the diameter of the laser induced wound after 1 h recovery (right). Data are from at least 25 first day adult animals. ∗∗∗∗p < 0.0001 by unpaired *t*-test. Error bars are SEM. Scale bar 10 μm. d) Representative fluorescence micrographs (left) and quantification of the fluorescence intensity of the ATFS-1:GFP reporter in hypodermal cell nuclei before and after 1 h laser induced wound. Data are from at least 65 first day adult animals. ∗p < 0.05; ∗∗∗∗p < 0.0001 by unpaired *t*-test. Error bars are SEM. Scale bar 20 μm.

Reduced body size is a well characterized phenotype associated with impaired mitochondrial function [59], and this trait is also observed in *gsr-1a trxr-2* double mutants. In *C. elegans*, body size is primarily regulated by the BMP/DBL-1 signaling pathway, which promotes endoreduplication of hypodermal cells and transcriptional control of collagen genes, the major structural components of worm cuticle, synthesized by the underlying hypodermis [[90], [91], [92]]. Interestingly, our RNAseq analysis revealed upregulation of genes encoding collagens and other proteins involved in cuticle morphogenesis (Fig. 3b and Supplementary Tables 2 and 3). Notably, several of these genes, including *lon-3* and *sqt-1*, function downstream BMP/DBL-1 pathway to control worm body size [93,94]. As elevated *lon-3* expression is known to decrease body size [95], we hypothesized that increased *lon-3* activity contributes to the reduced size phenotype observed in *gsr-1a trxr-2* animals. To test this, we used the *lon-3(sp23)* loss-of-function mutation [95] and found that the increased ratio in body size of the *gsr-1a trxr-2; lon-3* triple mutant compared to *gsr-1a trxr-1* control was similar to that of *lon-3* single mutant compared to wild-type control (Fig. 7a), indicating that *lon-3* is not responsible for the small phenotype. Furthermore, *gsr-1a trxr-2* double mutants have the same number of nuclei in the hypodermal hyp-7 syncytium as wild-type animals (Fig. 7b), suggesting that neither defective cuticle formation nor impaired hypodermal DNA content (ploidy) accounts for the reduced body size of *gsr-1a trxr-2* worms. Given the high number of collagen encoding genes present in the *C. elegans* genome [91], we cannot rule out that redox regulation of collagens other than *lon-3* may control body size in the double mutants.

Hypodermal wounding induces a distinct innate immune response initiated by a mitochondrial ROS production at the site of injury, triggering a rapid and reversible mitochondrial fragmentation that depends on cytoplasmic calcium and the mitochondrial Rho GTPase MIRO-1 [39,96,97]. As *gsr-1a trxr-2* double mutants induce an immune response at the transcriptional level and also show increased mitochondrial fragmentation in hypodermis, we next asked whether wound repair dynamics is altered in these animals. Indeed, laser-wounding experiments revealed that *gsr-1a trxr-2* worms repair wounds more rapidly than wild-type controls, as measured by contraction of the actin right at the injury site (Fig. 7c). Additionally, wounding triggered nuclear translocation of an ATFS-1:GFP fusion protein in hypodermal cells, a phenotype that is also found in unwounded *gsr-1a trxr-2* double mutants (Fig. 7d). Together, these observations suggest that accelerated wound repair in *gsr-1a trxr-2* mutants may be driven by chronic activation of ATFS-1-dependent stress signaling in the hypodermis.

**Fig. 8:**
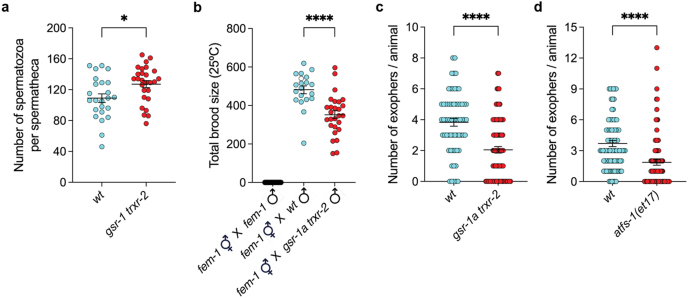
**Fertilization capacity and exopher production phenotypes in *gsr-1a(syb266) trxr-2(tm2047)* mutants.** a) Quantification of the number of spermatozoa per spermatheca. Data are from four independent experiments with at least four spermathecas per assay. ∗p < 0.05 by unpaired *t*-test. Error bars are SEM. b) Quantification of the total brood size from *fem-1(hz17)* hermaphrodites fertilized at 25 °C. Data are from three independent experiments including at least 10 crosses and progeny production was monitored over eleven days. ∗∗∗∗p < 0.0001 by unpaired *t*-test. Error bars are SEM. c,d) Quantification of muscle exopher formation in worms expressing a TOMM-20(1-50):GFP reporter under the control of the *myo-3* promoter. Data are from three independent experiments with at least ten animals per assay. ∗∗∗∗p < 0.0001 by two-tailed Mann-Whitney test. Error bars are SEM.

*gsr-1a trxr-2* double mutants have decreased progeny production, a phenotype previously associated with impaired mitochondrial function in *C. elegans* [60]. Because our RNAseq analysis revealed an upregulation of genes involved in male gamete generation and function (Fig. 3b), we first examined sperm production and fertilization capacity in *gsr-1a trxr-2* animals. Although *gsr-1a trxr-2* double mutants produced a comparable number of spermatozoa as compared to wild-type controls, their fertilization efficiency was markedly reduced (Fig. 8a and b). To evaluate whether maternal mechanisms also account to the lower progeny of *gsr-1a trxr-2* double mutants we next quantified exopher production in these animals. As described previously, exophers represent a specialised class of exceptionally large extracellular vesicles that enable *C. elegans* cells to externalise material that cannot be efficiently processed by conventional intracellular quality-control pathways. First described in neurons as a mechanism for expelling damaged mitochondria and protein aggregates [9], exophers are also produced by body-wall muscle, where they serve dual functions: the removal of mitochondria and the export of yolk proteins to support embryonic development [10]. As shown in Fig. 8c, *gsr-1a trxr-2* double mutants generate fewer muscle-derived exophers compared to wild-type controls. Given the induction of UPR^mt^ in *gsr-1a trxr-2* animals, we next examined whether constitutive activation of ATFS-1 influences exopher formation. In *atfs-1(et17)* animals, which express an ATFS-1 variant with a defective mitochondrial targeting sequence leading to constitutive ATFS-1 nuclear localization, the number of muscle exopher formation was also reduced (Fig. 8d). This finding provides a potential mechanistic link between disrupted mitochondrial redox homeostasis and impaired exopher formation that may reduce yolk proteins provision to the developing embryos, perhaps by shifting reproductive resources to mitochondrial repair. However, as embryo-derived factors induce the production of exophers [10], we cannot rule out that a reduced number of embryos in the uterus of *gsr-1a trxr-2* double mutants as a consequence of their reproductive system phenotypes (Fig. 1e–g) may also contribute to the lower exopher production in these animals. Taken together, our data suggest that the diminished progeny production in *gsr-1a trxr-2* animals can result from both reduced sperm fertilization capacity and decreased muscle-derived exopher formation.

In summary, while disruption of mitochondrial redox homeostasis compromises muscle and sperm function, it may simultaneously promote protective adaptations in other tissues, such as hypodermis.

## 4 Discussion

Mitochondria rely on the import of reduced glutathione from the cytoplasm, where it is synthesized. Accordingly, mammalian cells lacking the mitochondrial GSH importer paralogues SLC25A39 and SLC25A40 fail to proliferate [57], and this phenotype is conserved in *C. elegans* as null mutants of the *C16C10.1* gene, the single worm orthologue of SLC25A39/40 genes, die at L1 stage. In *C. elegans*, deletion of *gsr-1* first exon selectively abolishes the expression of the mitochondrial GSR-1a isoform while maintaining the expression of the cytoplasmic isoform GSR-1b, which is essential for survival [18]. Interestingly, *gsr-1a* mutants exhibit minimal transcriptional changes, similarly to *trxr-2* mutants lacking mitochondrial thioredoxin reductase. In contrast, the *gsr-1a trxr-2* double mutant has a robust alteration of its transcriptome, indicating that, like in the cytoplasm, these two redox systems act redundantly to maintain mitochondrial redox homeostasis.

Because the primary function of glutathione reductase is to regenerate GSH from GSSG, the observed transcriptional reprogramming in *gsr-1a trxr-2* double mutants suggests that the mitochondrial thioredoxin system may substitute this GSSG recycling function in the absence of mitochondrial GSR-1a. The viability of *gsr-1a trxr-2* animals, with only modest physiological defects, and the maintenance of a normal GSH/GSSG ratio in whole animal cell cytoplasm and muscle cell mitochondria, could be explained by the existence of a third GSSG reduction system in mitochondria either directly by dihydrolipoic acid or via mitochondrial glutaredoxin GLRX-5 and dihydrolipoamide [98,99]. Alternatively, an active GSSG export mechanism may operate in these animals to reduce it in the cytoplasm. Indeed, *C. elegans* mutants lacking the putative GSSG exporter *abtm-1* are lethal [100]. Together, it is then plausible that mitochondrial thioredoxin reductase and glutathione reductase systems are more involved in general redox homeostasis maintenance rather than recycling mitochondrial GSSG, which can then be performed in the cytoplasm if efficiently exported from mitochondria or within mitochondria through the lipoic acid cycle.

The transcriptional signature of the *gsr-1a trxr-2* double mutant resembles that of other mutants that impair core mitochondrial functions, including genes involved in stress resistance and immune response [65,66,72,73]. Consistent with this, *gsr-1a trxr-2* animals are smaller and display germline developmental defects that reduce progeny production and extend the egg-laying period, phenotypes associated with impaired mitochondrial function [59,74]. Related to this, and specific to mitochondrial impairment, mitohormesis is an adaptive response by which a mild, sublethal mitochondrial stress triggers a number of cellular changes leading to a rewiring of cell metabolism, protein folding capacity and redox environment that collectively predispose the organism to be less vulnerable to subsequent more severe stresses (reviewed in Ref. [101]). However, despite *gsr-1a trxr-2* double mutants are small and have reduced progeny, phenotypes shared by mitohormetic worms [102,103], they do not show enhanced resistance to pathogen infection or toxicants exposure, do not live longer and exhibit normal ROS levels and basal respiration. Thus, disruption of mitochondrial redox homeostasis alters mitochondrial function but does not seem to elicit a beneficial adaptive mitohormetic response.

*gsr-1a trxr-2* animals trigger a transcriptional response consistent with the hypothesis that maintenance of redox homeostasis is a core mitochondrial function, as its impairment triggers the expression of many genes that are *bona-fide* targets of ATFS-1, the master regulator of UPR^mt^ [62]. To our knowledge, this genetic background induces one of the strongest constitutive ATFS-1 nuclear import phenotypes described to date. These findings highlight the central role of mitochondrial redox balance in sustaining proteostasis and demonstrate the essential role of ATFS-1 activation and UPR^mt^ induction during development when mitochondrial redox homeostasis is disrupted, proven by the lethal phenotype of a *gsr-1a trxr-2; atfs-1* triple mutant.

Throughout development, *C. elegans* undergoes a mitochondrial network expansion program, particularly critical during germline maturation at the L3 to L4 larval stage transition, which requires ATFS-1 mediated UPR^m^ induction [104,105]. Our data support the hypothesis that a tightly regulated mitochondrial redox function is essential for a correct germline development and acquisition of normal size and that the constitutive UPR^mt^ induction in *gsr-1a trxr-2* animals can only partly restore mitochondrial functionally to compensate germline and size defects. This partial compensation likely underlies the reduced body size, impaired gonad migration and decreased progeny phenotypes observed in the double mutants, reflecting a trade-off between stress mitigation and reproductive fitness, which is dependent on ATFS-1 and UPR^mt^ [106].

We further found that mitochondrial dynamics in *gsr-1a trxr-2* double mutants is regulated in a tissue-specific manner, with muscle cells showing increased elongated mitochondria while hypodermal cells presenting a high degree of mitochondrial fragmentation. Proper mitochondrial dynamics is essential for muscle performance, as illustrated by the motility defects described in mutants that either disrupt mitochondrial fusion (*fzo-1*) or mitochondrial fission (*drp-1*) [107]. In this context, *gsr-1a trxr-2* double mutants also have impaired motility phenotypes and other muscle related defects such as reduced pharyngeal pumping and, more strikingly, display severe developmental delays when combined with mutations in *drp-1* or *fzo-1* genes, indicating that mitochondrial redox imbalance sensitizes mitochondrial dynamics. In contrast, the small increase of mitophagy in *gsr-1a trxr-2* double mutants does not have any impact on development, demonstrating that disruption of mitochondrial redox homeostasis primarily alters mitochondrial remodeling rather than causing accumulation of damaged mitochondria.

Interestingly, calcium influx into the mitochondrial matrix, which is important to provide energy for muscle contraction, is not altered under basal conditions in *gsr-1a trxr-2* animals, but it is strongly decreased upon carbachol treatment, that induces a massive calcium influx through the plasma membrane and the subsequent buffering by import into mitochondria [87,89]. Our data suggest that MCU-1 inner mitochondrial membrane Ca^2+^ uniporter, which mediates calcium import into mitochondrial matrix upon carbachol treatment [108], is negatively regulated when mitochondrial redox homeostasis is disrupted. The fact that mammalian MCU is activated by glutathionylation in a conserved cysteine residue [109], also present in *C. elegans*, may provide a plausible explanation for the lower Ca^2+^ import in *gsr-1a trxr-2* double mutants as MCU-1 glutathionylation may be defective in muscle cells.

In contrast to the increased elongation of the mitochondria network in muscle cells, hypodermal cells have a much more fragmented network in *gsr-1a trxr-2* worms as compared to wild-type animals. Unexpectedly, *gsr-1a trxr-2* animals are more efficient repairing laser-induced lesions in the hypodermis, which may appear as a counterintuitive result considering that closure of hypodermal wounds has been shown to be dependent on Ca^2+^ import into the mitochondria via MCU-1 [39]. One possible explanation for this apparently contradictory result compared to muscle cells is that laser-wounding, a much more damaging treatment than carbachol exposure, may also induce an increase in cytoplasmic GSH import into mitochondria to sustain MCU-1 activity, as it has been reported following traumatic brain injury in mice [110]. Alternatively, enhanced mitochondrial fragmentation in hypodermis may induce MCU-1 levels in this tissue, while mitochondrial elongation may decrease MCU-1 expression in muscle cells. It will be interesting to determine whether ATFS-1 directly regulates cytoskeletal repair genes or modulates calcium dynamics during wound closure.

In addition to their roles in somatic tissues, mitochondria are essential regulators of *C. elegans* spermatogenesis and sperm activation, the transition from immotile spermatids to fully functional, motile spermatozoa [111,112]. Notably, *gsr-1a trxr-2* double mutants exhibit reduced fertilization capacity, even though they upregulate multiple genes required for the formation of functional gametes as shown by transcriptomic analysis. How defects in mitochondrial redox homeostasis specifically impair sperm maturation and/or sperm function, and whether diminished muscle-derived exopher yolk provisioning to developing embryos contributes to the reduced brood-size phenotype in these mutants, remain important open questions that deserve further investigation.

In conclusion, in this work we provide a genetic and functional characterization a *C. elegans* mutant with impaired mitochondrial redox homeostasis, which leads to mild but pleiotropic phenotypes. The availability of this mutant simultaneously lacking mitochondrial thioredoxin and glutathione reductases offers a valuable model to investigate how mitochondrial redox control influences key cellular and organismal functions. Future studies can build on this work aiming to identify the mitochondria-derived signal responsible for constitutive ATFS-1 activation in *gsr-1a trxr-2* mutants or to examine how endoplasmic reticulum dynamics adapt to compromised mitochondrial redox status. Together, this genetic model provides a useful platform for advancing our understanding of *in vivo* redox regulation in mitochondria.

## Data availability statement

RNAseq raw data are deposited at the Sequence Read Archive of NCBI with the ID number GSE311484.

## CRediT authorship contribution statement

**Marina Valenzuela-Villatoro:** Conceptualization, Data curation, Formal analysis, Investigation, Methodology. **Patricia de la Cruz-Ruiz:** Conceptualization, Data curation, Formal analysis, Investigation, Methodology, Writing – original draft. **David Guerrero-Gómez:** Formal analysis, Investigation, Methodology. **Eva Gómez-Orte:** Formal analysis, Investigation, Methodology. **Alfonso Schiavi:** Formal analysis, Investigation, Methodology. **Silvia Maglioni:** Formal analysis, Investigation, Methodology. **Mayte Montero:** Formal analysis, Investigation, Methodology. **Rosalba I. Fonteriz:** Formal analysis, Investigation, Methodology. **José Carlos Casas-Martínez:** Data curation, Formal analysis, Investigation, Methodology. **Nicole E. Briand:** Investigation, Methodology. **Jingxiu Xu:** Investigation, Methodology. **María Jesús Rodriguez-Palero:** Formal analysis, Investigation, Methodology. **Marta Artal-Sanz:** Investigation, Methodology. **Katarzyna Olek:** Investigation, Methodology. **Justyna Polaczyk:** Investigation, Methodology. **Michal Turek:** Investigation, Methodology. **Wojciech Pokrzywa:** Investigation, Methodology. **Suhong Xu:** Investigation, Methodology. **Javier E. Irazoqui:** Formal analysis, Investigation, Methodology. **Brian McDonagh:** Investigation, Methodology, Writing – review & editing. **Javier Álvarez:** Investigation, Methodology, Writing – review & editing. **María Olmedo:** Formal analysis, Investigation, Methodology. **Natascia Ventura:** Formal analysis, Investigation, Methodology. **Juan Cabello:** Conceptualization, Investigation, Methodology, Writing – review & editing. **Antonio Miranda-Vizuete:** Conceptualization, Data curation, Formal analysis, Funding acquisition, Project administration, Supervision, Writing – original draft.

## Declaration of competing interest

Dr. Antonio Miranda-Vizuete, corresponding author of the manuscript entitled “Mitochondrial redox homeostasis links organellar stress surveillance to germline and somatic integrity in *Caenorhabditis elegans*” and on behalf of all authors, declares that have no known competing financial interests or personal relationships applicable to the work reported in this paper.
