# Supplementary material for: Mitochondrial redox homeostasis links organellar stress surveillance to germline and somatic integrity in Caenorhabditis elegans
Source: Redox Biol. 2026 Mar 9;93:104115. doi: 10.1016/j.redox.2026.104115 (PMC13099523; doi:10.1016/j.redox.2026.104115)
Supplement: Multimedia component 6 [file mmc6.pdf]

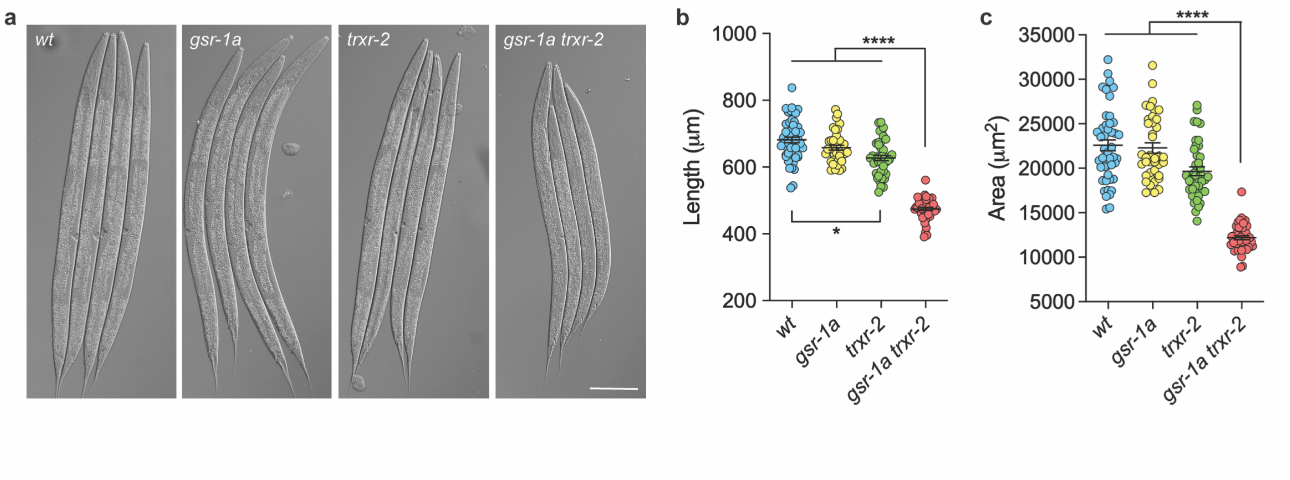

Supplementary Figure 1. Size phenotypes of *gsr-1a(syb266)* and *trxr-2(tm2047)* mutants. a) Representative micrographs of animals of the specified genotypes at second day after synchronized egg-lay. Scale bar 100 µm. Quantification of b) worm length and c) worm area. Data are from two independent experiments with at least 25 animals per assay. Error bars are SEM. *p<0.05; **** p<0.0001 by Kruskal-Wallis test.

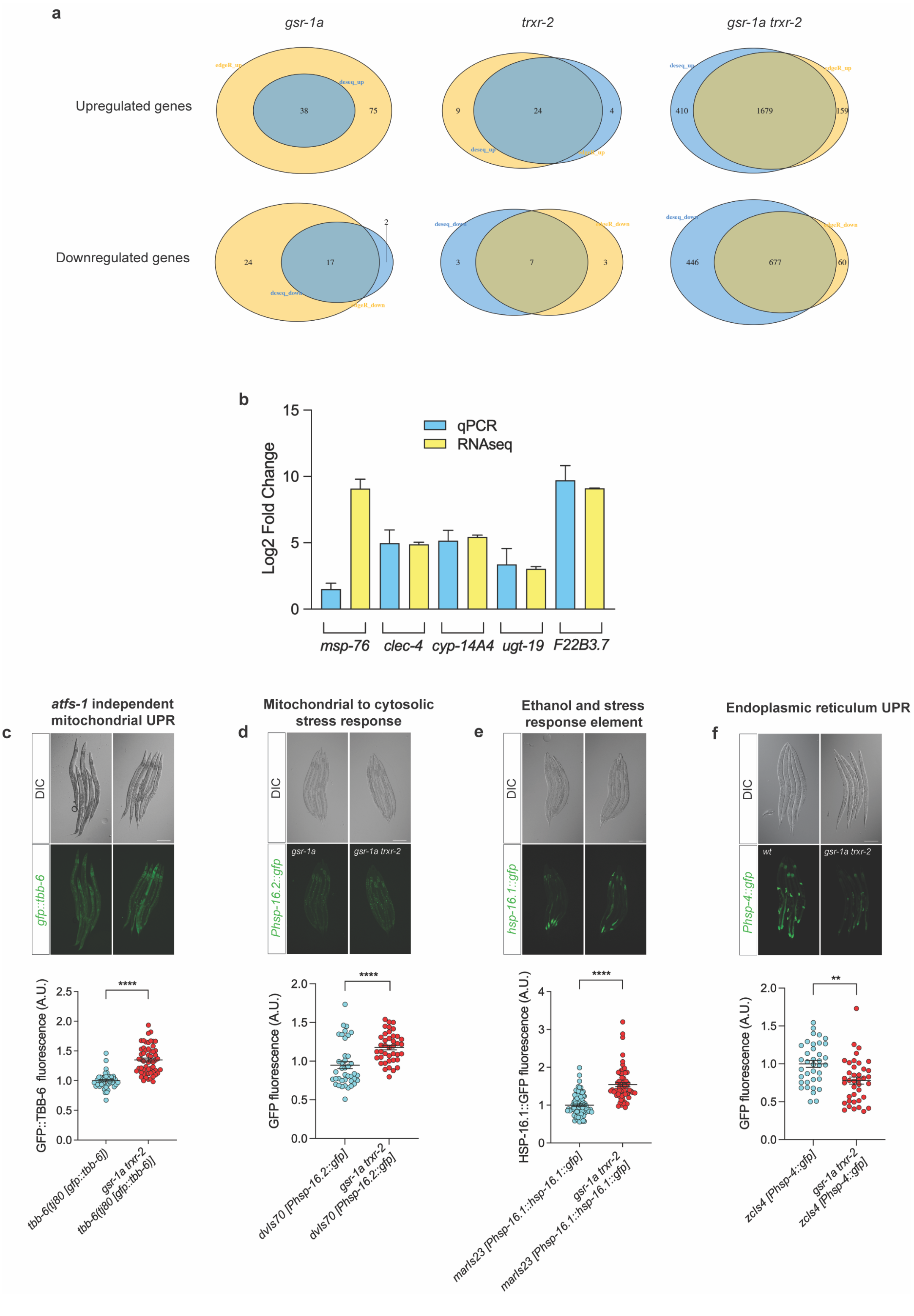

Supplementary Figure 2. Transcriptomic analysis and stress response markers in *gsr-1a(syb266)* and *trxr-2(tm2047)* mutants. a) Venn diagrams and overlap of DEGs analysed by edgeR and Deseq2 packages (Robinson et al. 2010; Love et al. 2014). b) Comparison of selected DEGs by RNAseq and qPCR analysis. c-f) Representative differential interference contrast and fluorescence micrographs and quantification of worms at first day of adulthood, except *dvIs70* animals at L4 stage, expressing reporters for c) *atfs-1* independent UPR^{mt} (Munkacsy et al. 2016); d) mitochondrial-to-cytosolic stress response (Kim et al. 2016); e) ethanol and stress response element (Tjahjono et al. 2020) and f) UPR^{er} (Calfon et al. 2002). Data are from three different experiments with at least 10 animals per assay. Error bars are SEM. ** p<0.01; **** p<0.0001 by unpaired t-test. Scale bar 200 μm.

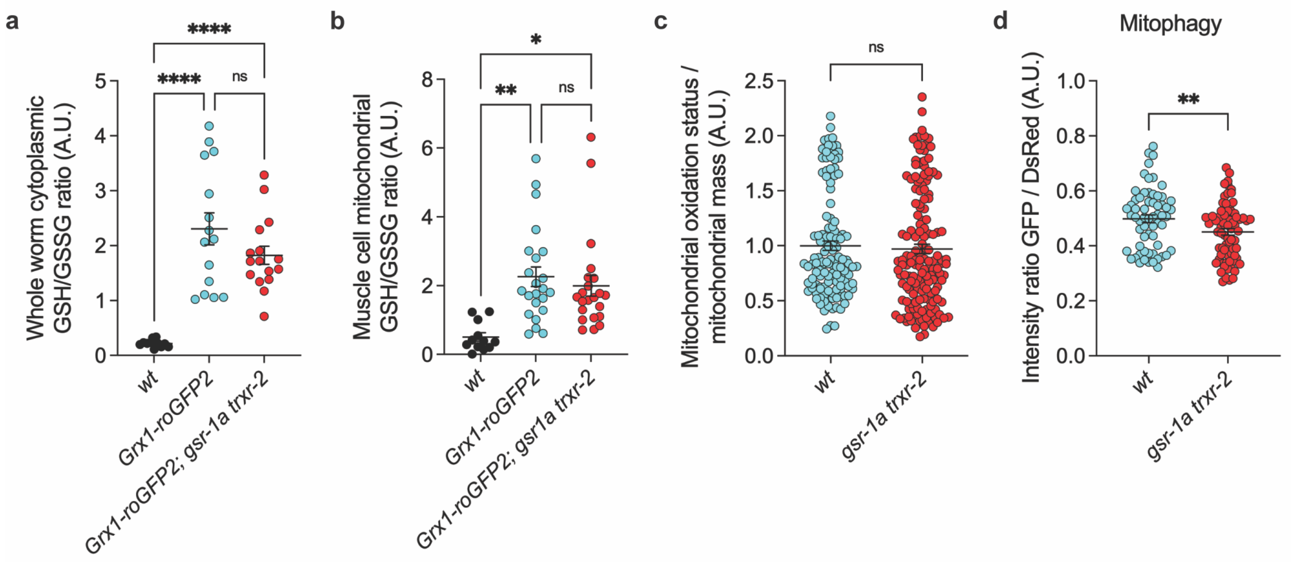

Supplementary Figure 3. Glutathione redox status, mitochondrial matrix oxidation and mitophagy in *gsr-1a(syb266) trxr-2(tm2047)* mutants. Quantification of a) GSH/GSSG ratio using a whole worm cytoplasmic Grx1-roGFP2 biosensor (Back et al. 2012) , b) GSH/GSSG ratio using a muscle cell mitochondrial Grx1-roGFP2 biosensor (Guo et al. 2023), c) mitochondrial matrix oxidation status using MitoTracker™ Red CM-H_2XRos and d) mitophagy using a mtRosella biosensor (Palikaras et al. 2015). All data are from three independent experiments with first day adult animals using approximately 250 worms. ns, not significant and ****p < 0.0001 by ordinary One-way ANOVA (a,b); at least 40 worms. ns, not significant by two-tailed Mann–Whitney test (c), and at least 10 worms. **p<0.01 by unpaired t-test. Represented is the ratio between pH-sensitive GFP to pH-insensitive DsRed (d). Error bars are SEM.

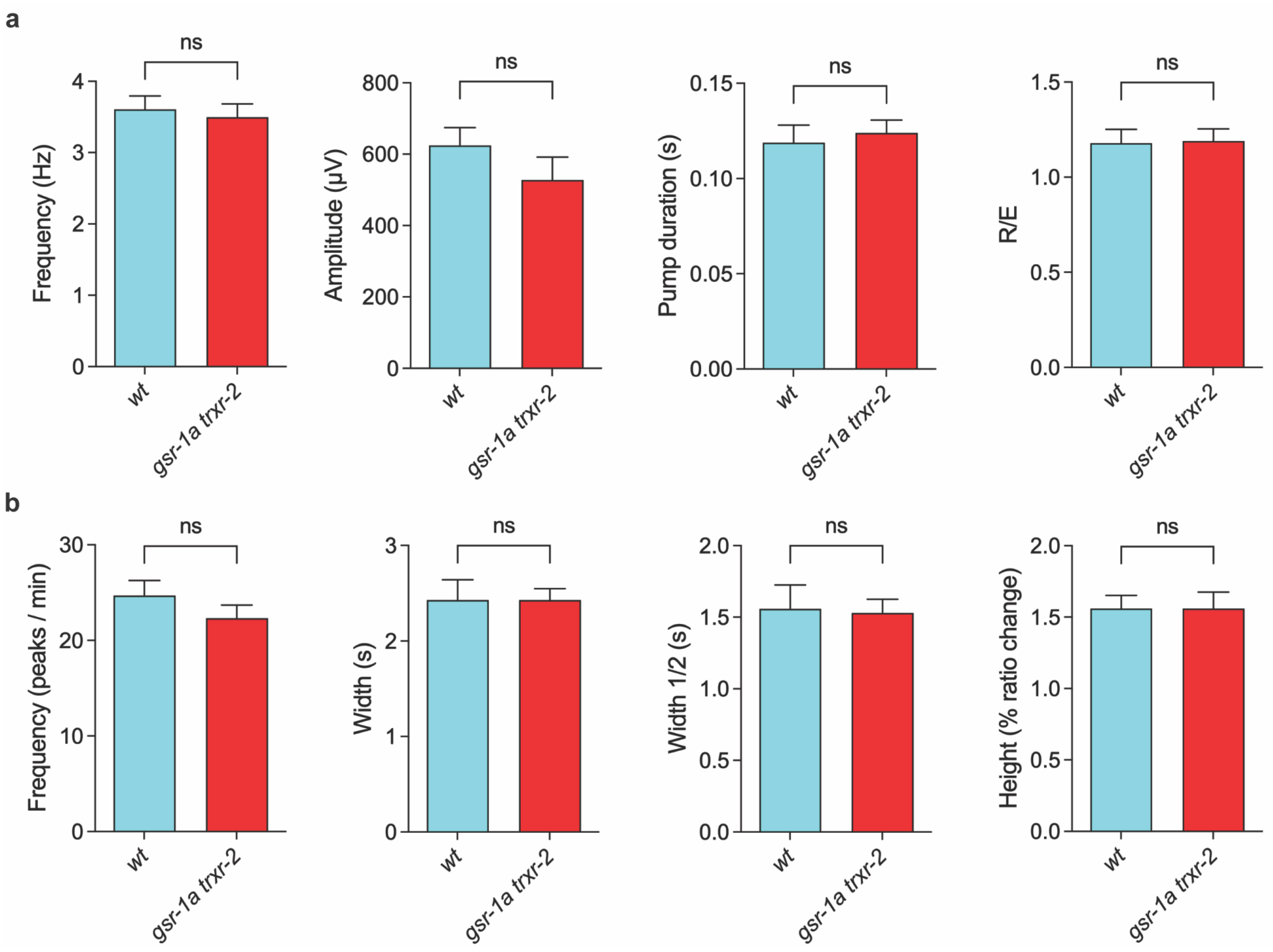

Supplementary Figure 4. Electropharyngeogram and basal mitochondrial calcium uptake parameters in wild type and *gsr-1a trxr-2* mutants. a) Quantification of electropharyngeogram frequency, pump duration, amplitude and repolarization (R) / depolarization (E) ratio. Data are the mean +/- SEM from experiments performed in 31 wild-type worms and 30 *gsr-1a trxr-2* double mutants. ns, not significant by unpaired t-test. b) Quantification of basal mitochondrial Ca^{2+} peaks: frequency, width at baseline (width), width at half-height (width ½) and height. Data are the mean +/- SEM from experiments performed in 24 wild-type worms and 32 *gsr-1a trxr-2* double mutants. ns, not significant by unpaired t-test.

REFERENCES

Back, P., W.H. De Vos, G.G. Depuydt, F. Matthijssens, J.R. Vanfleteren *et al.*, 2012 Exploring real-time in vivo redox biology of developing and aging Caenorhabditis elegans. *Free Radic Biol Med* 52 (5):850-859.

Calfon, M., H. Zeng, F. Urano , J.H. Till, S.R. Hubbard *et al.*, 2002 IRE1 couples endoplasmic reticulum load to secretory capacity by processing the XBP-1 mRNA. *Nature* 415 (6867):92-96.

Guo, M., X. Qiao, Y. Wang, Z.H. Li, C. Shi *et al.*, 2023 Mitochondrial translational defect extends lifespan in C. elegans by activating UPR(mt). *Redox Biol* 63:102722.

Love, M.I., W. Huber, and S. Anders, 2014 Moderated estimation of fold change and dispersion for RNA-seq data with DESeq2. *Genome Biol* 15 (12):550.

Kim, H.E., A.R. Grant, M.S. Simic, R.A. Kohnz, D.K. Nomura *et al.*, 2016 Lipid Biosynthesis Coordinates a Mitochondrial-to-Cytosolic Stress Response. *Cell* 166 (6):1539-1552 e1516.

Munkacsy, E., M.H. Khan, R.K. Lane, M.B. Borror, J.H. Park *et al.*, 2016 DLK-1, SEK-3 and PMK-3 Are Required for the Life Extension Induced by Mitochondrial Bioenergetic Disruption in C. elegans. *PLoS Genet* 12 (7):e1006133.

Palikaras, K., E. Lionaki, and N. Tavernarakis, 2015 Coordination of mitophagy and mitochondrial biogenesis during ageing in C. elegans. *Nature* 521 (7553):525-528.

Robinson, M.D., D.J. McCarthy, and G.K. Smyth, 2010 edgeR: a Bioconductor package for differential expression analysis of digital gene expression data. *Bioinformatics* 26 (1):139-140.

Tjahjono, E., A.P. McAnena, and N.V. Kirienko, 2020 The evolutionarily conserved ESRE stress response network is activated by ROS and mitochondrial damage. *BMC Biol* 18 (1):74.
